# A Suppressor/Avirulence Gene Combination in *Hyaloperonospora arabidopsidis* Determines Race Specificity in *Arabidopsis thaliana*

**DOI:** 10.3389/fpls.2018.00265

**Published:** 2018-03-01

**Authors:** Alison Woods-Tör, David J. Studholme, Volkan Cevik, Osman Telli, Eric B. Holub, Mahmut Tör

**Affiliations:** ^1^Institute of Science and the Environment, University of Worcester, Worcester, United Kingdom; ^2^College of Life and Environmental Sciences, University of Exeter, Exeter, United Kingdom; ^3^Department of Biology and Biochemistry, University of Bath, Bath, United Kingdom; ^4^Warwick Crop Centre, School of Life Sciences, University of Warwick, Coventry, United Kingdom

**Keywords:** effector, avirulence inhibitor, suppressor, oomycete, *Arabidopsis*, downy mildew, heterozygosity

## Abstract

The pathosystem of *Arabidopsis thaliana* and diploid biotrophic oomycete *Hyaloperonospora arabidopsidis* (*Hpa*) has been a model for investigating the molecular basis of Flor's gene-for-gene hypothesis. The isolates *Hpa*-Noks1 and *Hpa*-Cala2 are virulent on *Arabidopsis* accession RMX-A02 whilst an F_1_ generated from a cross between these two isolates was avirulent. The F_2_ progeny segregated 3,1 (avirulent, virulent), indicating a single major effect *AVR* locus in this pathogen. SNP-based linkage mapping confirmed a single *AVR* locus within a 14 kb map interval containing two genes encoding putative effectors. The *Hpa*-Cala2 allele of one gene, designated *H*. *a**rabidopsidis* *c**ryptic1* (*HAC1*), encodes a protein with a signal peptide and an RxLR/dEER motif, and triggers a defense response in RMX-A02. The second gene is heterozygous in *Hpa*-Cala2. One allele, designated *S**uppressor of* *HAC1*^*Cala2*^ (*S-HAC1*^*Cala2*^) encodes a protein with a signal peptide and a dKEE motif with no RxLR motif; the other allele (*s-hac1* ^*Cala2*^) encodes a protein with a signal peptide, a dEEE motif and is divergent in sequence from the *S-HAC1*^*Cala2*^ allele. In selfed progeny from *Hpa*-Cala2, dominant *S-HAC1*^*Cala2*^ allele carrying progeny correlates with virulence in RMX-A02, whereas homozygous recessive *s-hac1*^*Cala2*^ carrying progeny were avirulent. Genetic investigations suggested other heterozygous suppressor loci might exist in the *Hpa*-Cala2 genome.

## Introduction

Oomycetes are diploid microorganisms that resemble fungi in morphology and lifestyle, having filamentous mycelia and developing specialized structures such as haustoria during infection. However, they are taxonomically distinct from fungi and are instead placed within the Stramenopiles (Keeling et al., 2005). Oomycetes contain several economically important crop pathogens including *Phytophthora infestans* (tomato and potato late blight), *P. ramorum* (sudden oak death), *Plasmopora viticola* (grapevine downy mildew), *Pythium ultimum* (damping off and root rot), *Bremia lactuca* (lettuce downy mildew), and *Albugo candida* (white blister rust of crucifers) (Kamoun et al., 2015).

The biotrophic oomycete *Hyaloperonospora arabidopsidis* (*Hpa*, formerly known as *H. parasitica*) has co-evolved as a downy mildew pathogen in wild populations of *Arabidopsis thaliana* (Göker et al., 2004; Holub, 2006), and has been used for more than 25 years as an experimental model (Koch and Slusarenko, 1990; Tör et al., 1994) for investigating the molecular basis of the gene-for-gene theory proposed by H. H. Flor to explain genetic evidence that for each gene controlling resistance in the host, there is a corresponding gene controlling pathogenicity in the pathogen (Flor, 1946). At least seven downy mildew *R*-genes have been characterized in this pathosystem (Botella et al., 1998; McDowell et al., 1998; Bittner-Eddy et al., 1999; van der Biezen et al., 2002; Sinapidou et al., 2004; Eulgem et al., 2007), and all encode cytoplasmic NLR proteins that contain a conserved nucleotide-binding site (NBS) and a variable leucine-rich repeat (LRR) domain. Three of the seven predicted *AVR* elicitors have also been characterized (Allen et al., 2004; Rehmany et al., 2005; Bailey et al., 2011) and all encode secreted effector-like proteins.

Plant pathogens deliver effectors to establish infection by suppressing the host immune system, altering plant physiology, and colonizing the host cell (Doehlemann and Hemetsberger, 2013; Win et al., 2013). Current consensus suggests that effectors target apoplastic and cytoplasmic components (Shan et al., 2008; Dodds and Rathjen, 2010; Pedersen et al., 2012). Fungal and oomycete pathogens secrete effectors with the involvement of an N-terminal signal peptide (Kamoun, 2006; Song et al., 2009), and some studies of oomycetes suggest that RxLR and dEER motifs may be involved in the translocation of these effectors into the host cell (Whisson et al., 2007; Tyler et al., 2013). However, a recent proteomic investigation on the RxLR effector AVR3a from the potato pathogen *P. infestans* demonstrated that the N-terminal part of the native protein up to and including the RxLR motif but excluding the dEER motif is cleaved off before the secretion, indicating a possible role of the RxLR motif in secretion of the effector from the pathogen (Wawra et al., 2017). Effectors that elicit a defense response due to detection by an R-protein are referred to as avirulence elicitor proteins (Jones and Dangl, 2006).

Interestingly, H. H. Flor described an exception to the simplified “gene-for-gene” model in which avirulent progeny of the flax rust fungus *Melampsora lini* were derived from sexual mating of two virulent isolates. He used selfing, back-crossing, and inter-mating of progeny to verify that several avirulence genes each had a matching suppressor/inhibitor gene. Lawrence et al. (1981) subsequently confirmed these results in the flax rust pathosystem. A similar example was described from detailed genetic studies in the rice blast fungus *Magnaporthe grisea* (Ellingboe, 1992; Lau et al., 1993). In some cases, the suppressor and the avirulent determinant were tightly linked on the same chromosome and could not be separated by recombination (Lau et al., 1993). Recent studies have begun to reveal the molecular basis of such interactions in other fungi. For example, Avr1 of *Fusarium oxysporum* f.sp. *lycopercisi* is recognized by the tomato *R*-gene product I-1 but suppresses the recognition of Avr2 and Avr3 by I-2 and I-3, respectively (Houterman et al., 2008). Current studies suggest that the suppressor/avirulence gene combination provides the basis of specificity in fungi and oomycetes (Bourras et al., 2016).

Whilst genetic studies into *Arabidopsis-Hpa* interactions have shown the existence of a gene-for-gene relationship, molecular, and bioinformatics investigations have helped the cloning of several avirulent determinants in the pathogen and the corresponding *R*-genes in the host (Holub et al., 1994; Holub, 2006; Baxter et al., 2010; Bailey et al., 2011). Some of the *Hpa* RXLR-type effector proteins have been molecularly characterized. For example, ATR1 (Rehmany et al., 2005) is recognized by RPP1 by direct association via C-terminal leucine-rich repeats (Steinbrenner et al., 2015). ATR13 (Allen et al., 2004) is a highly polymorphic and dynamic protein with two surface-exposed patches of polymorphism, only one of which is involved in specific recognition of RPP13-Nd (Leonelli et al., 2011). ATR39-1 has been identified by computational methods and is recognized by RPP39 in *Arabidopsis* accession Weiningen (Wei-0) (Goritschnig et al., 2012). Recently we cloned ATR5^Emoy2^, which does not have the RxLR motif but contains a dEER motif (Bailey et al., 2011). All of these *Hpa* effectors are avirulence determinants and, until now, no suppressor has been identified for any of them. Here, we report the genetic identification and map-based cloning of a *H*. *a**rabidopsidis* *c**ryptic* (*HAC1*^*Cala2*^) avirulence determinant and a predicted matching suppressor (*S-HAC1* ^*Cala2*^) from the same interval using a sexual cross between two virulent isolates of *H. arabidopsidis*. HAC1^Cala2^ is an RxLR-dEER type putative effector protein inherited in progeny from the parent isolate *Hpa*-Cala2 and can trigger an immune response in the *A. thaliana* accession RMX-A02. *S-HAC1* ^*Cala2*^ is in the same parent isolate within the genetic interval next to the *HAC1*^*Cala2*^ gene and encodes a putative effector protein with a dKEE motif. S-HAC1 ^Cala2^ is epistatic over HAC1^Cala2^.

## Materials and methods

### *Arabidopsis* germplasm

*A. thaliana* accessions Columbia (Col-0), Landsberg *erecta* (Ler-0) and a subset from the Nordborg-Bergelson collection (Nordborg et al., 2005) were used in this study. All can be obtained from the Nottingham Arabidopsis Stock Center (http://arabidopsis.info). Wassilewskija-*eds1.1* was described previously (Parker et al., 1996).

### Pathogen isolates and pathology methods

All isolates of *H. arabidopsidis* were maintained on Ws-*eds1* (Parker et al., 1996). Generation of a cross (CaNo F_1_) between *Hpa*-Cala2 and *Hpa*-Noks1 was described previously (Bailey et al., 2011). Preparation of inoculum for experiments, and the assessment of sporulation were as described previously in Tör et al. (2002).

### Bulk segregant analysis using next generation sequencing

Laboratory mating of *H. arabidopsidis* and production of CaNo F_2_ isolates was described in our previous work (Bailey et al., 2011). The accession RMX-A02 was screened with 54 randomly selected CaNo F_2_ isolates. DNA was extracted separately from each individual F_2_ isolate, and DNA from fifteen virulent (*hac1/hac1*) and fifteen avirulent (*HAC1*/±) F_2_ isolates was pooled in equal concentrations to make up the virulent and avirulent bulks, respectively. One lane of 100 bp paired-end Illumina HiSeq2500 sequencing data was generated from each bulked pool, comprising 104 million reads for the virulent bulk and 110 million reads for the avirulent bulk. The Illumina reads were first trimmed based on their quality scores using Btrim (Kong, 2011) with a cut-off of 25 for average quality scores within a moving window of 5 bp. The minimum acceptable read length was 25 bp (that is, reads that were shorter than 25 bp after trimming were discarded). Other parameters for Btrim were set to default values. SPAdes v. 3.6.1; SSPACE v. 3.0 were then employed to *de novo* assemble both *Hpa*-Cala2 and *Hpa*-Noks1 genome sequences. We then Used BWA-mem to do the alignment of reads against the *Hpa*-Noks1 genome assembly as a reference to map sequence reads from both virulent and avirulent bulks and the frequency of major allele at 26,722 sites have been examined. The contig 23137 (GenBank, LLKM01000918) had the highest percentage of SNP hit representing the largest difference between the bulks and used for subsequent mapping.

### Map-based cloning of *HAC1* and *S-HAC1*

SNPs identified between the virulent and avirulent bulks using the genomic contig 23137 from *Hpa*-Noks1 as a reference were converted to CAPS markers using dCAPS (http://helix.wustl.edu/dcaps/dcaps.html) (Neff et al., 2002). They were then used to map the *HAC1* locus. Once linkage was confirmed, flanking markers were used to identify the *Hpa*-Emoy2 genomic Scaffold 41, and were also used for screening further F_2_ isolates to identify recombinants. A total of 190 F_2_ isolates were screened with the molecular markers. Recombinant CaNo F_2_ isolates from either side of the interval were used to narrow the interval down to 14 kb on the Scaffold 41, 298,000–312,000. This region was checked on Ensembl Protist site (http://protists.ensembl.org) for possible ESTs. There were two gaps in the interval and both were filled manually by long PCR and sequencing the products. All PCR amplifications for mapping were performed in a total volume of 20 μl containing 20 ng of genomic DNA, forward and reverse primers (Supplemental Table 1) each at 0.2 μM, BioMix Red (Bioline). The PCR reaction consisted of a first step at 94°C for 3 min followed by 35 cycles of 30 s denaturation at 94°C, 30 s annealing at 50–60°C (based on T_m_ of primers) and 1 min extension at 72°C. Finally, an extension step was carried out at 72°C for 5 min. A 3 μl sample of each reaction volume was loaded onto a 1.5% agarose gel to ascertain whether PCR amplification was successful. The remaining 10–15 μl of PCR reactions were digested with relevant restriction enzymes following manufacturer's instructions. Digested products of PCR amplicons were separated on a 2% agarose gel containing TAE buffer at 110 V for 2 h, and visualized under UV light after staining with GelRed.

### Expression analysis

Total RNA was isolated from uninfected (control) or infected with *Hpa*-Cala2, *Hpa*-Noks1, *Hpa*-Emoy2, at 1, 2, 3, 4, 5, 6, and 7 day post inoculation (dpi) using RNeasy plant mini kit (Qiagen Ltd., West Sussex, UK) according to manufacturer's instructions. RT-PCR was performed with gene specific or *Hpa-Actin* primers (Supplemental Table 1), using 0.5 μg of total RNA as template. The PCR product was separated on a 1.5% agarose gel and stained with ethidium bromide. The products were verified by sequencing.

For quantitative RT-PCR, total RNA was isolated from Ws-*eds1* seedlings infected with *Hpa*-Cala2 at 3, 4, and 7 dpi. Real-time PCR was carried out using gene specific primers for *HAC1*^*Cala2*^*, S-HAC1*^*Cala2*^ and *s-hac1*^*Cala2*^. The 10 μl reaction mix consisted of 40 ng of RNA, 5 μl SensiFAST Syber mix (SensiFAST™ SYBR® No-ROX One-Step Kit) 0.5 μM of each primer, 0.1 μl reverse transcriptase, 0.2 μl RNA inhibitor and DEPC water. PCR conditions were as follows, 45°C 20 min, 95°C for 2 min, followed by 10 cycles touchdown procedure; 95°C for 10 s, 1°C decrease in each annealing step of (68–59)°C for 15 s, 72°C 20 s; then 30 cycles of 95°C for 10 s, 59°C for 15 s 72°C for 20 s. PCR efficiency was detected as 98.7%. Relative abundance of *HAC1*^*Cala2*^*, S-HAC1*^*Cala2*^, and *s-hac1*^*Cala2*^to *Hpa-Actin* was calculated as 2^∧^-dCt (Livak and Schmittgen, 2001). Three samples were included for each analysis and the experiments were repeated three times.

### RACE and DNA sequencing

Total RNA was isolated from infected plant materials using RNeasy plant mini kit (Qiagen Ltd., West Sussex, UK) according to manufacturer's instructions. RACE was performed using the GeneRacer kit (Life Technologies Ltd, UK), following manufacturer's instructions using gene specific and nested primers (Supplemental Table 6). PCR regime and subsequent cloning was carried out as described by Bailey et al. (2011). PCR products or plasmid clones were sequenced using Mix2Seq Kit by Eurofins Genomics (Wolverhampton, UK).

### Quantification of pathogen biomass and high-resolution melt curve analysis

Samples for genomic DNA isolation were collected from infected seedlings 7 dpi. Fifteen seedlings from each genotype made up a sample and three replicates were used for each sample. DNA was isolated using CTAB method (Doyle, 1987) and the PCR was performed in a total volume of 25 μl containing 50 ng of gDNA, 12.5 μl of SybrGreen Mastermix (ABI, Carlsbad, California), *Hpa-Actin* or *At-Actin* primers (Supplemental Table 1) and water on a Roche LightCycle 480 device. PCR conditions were as follows, 95°C for 4 min, then 10 cycles touchdown of 95°C for 30 s, annealing temperature of 65°C, decreasing 1°C every cycle to 56°C, and extension at 72°C for 30 s. After 10 cycles of touchdown, a further 25 cycles of 95°C for 30 s, 60 °C for 30 s and 72°C for 30 s and a final extension at 72°C for 5 min were carried out. Relative abundance of *Hpa-Actin* to *At-Actin* was calculated as 2^∧^-dCt (Livak and Schmittgen, 2001). High-resolution melt curve analysis (HRM) was carried out using gene specific primers for *s-hac1*^*Cala2*^ (Supplemental Table 1). The 20 μl reaction mix consisted of 30 ng of DNA, 10 μl HRM mix (Bioline Sensifast), 250 nM of each primer and sterile distilled water. PCR conditions were as follows, 95°C for 2 min, followed by 8 cycles of 95°C for 5 s, 65°C for 10 s, 72°C 25 s; then 37 cycles of 95°C for 5 s, 58°C for 10 s 72°C for 25 s. This was followed by a melt cycle as follows, 95°C for 15 s, 40°C for 15 s, 70°C for 1 s, then increasing to 95°C with a ramp rate of 0.2°Cs^−1^ with 25 fluorescence data acquisitions per °C.

### Light microscopy

Seedlings of infected and non-inoculated controls were stained with a solution of phenol, lactic acid, glycerol, and water (1,1,1,1) supplemented with 1 mg/ml trypan blue, decolourised in chloral hydrate and visualized under a compound microscope according to a previously described method (Koch and Slusarenko, 1990).

### *Agrobacterium*-mediated transformation

Full-length *HAC1, S-HAC1, and s-hac1* candidate alleles were amplified from *Hpa*-Cala2 and *Hpa*-Noks1 genomic DNA using gene specific primers and cloned into pDONR/zeo vector (Life technologies) by Gateway cloning technology. Candidate genes were transferred to the binary vectors pEarleyGate100 (Earley et al., 2006) that has the constitutive 35S CMV promoter or pBAV150 (Vinatzer et al., 2006), which has Dexamethasone inducible promoter by LR recombination (Life technologies). The constructs were electroporated into *E. coli* strain DH10B and positive clones were identified by PCR and sequencing. The construct was then introduced into *Agrobacterium tumefaciens* strain GV3101 by electroporation and the RMX-A02 and Col-0 plants were transformed by the floral dip method (Clough and Bent, 1998). Transformants were selected by growing plants in soil soaked with 0.1% Basta (AgrEvo, Norfolk, UK) and inserts were confirmed by PCR using gene specific primers. Homozygous T_3_ plants were obtained and used for the subsequent experiments.

### Statistical analysis

For statistical analysis, two-tailed students *t*-tests were performed on data obtained in plant infection assays and qRT-PCR.

### Bioinformatics

IICB Genomics and Transcriptomics Resources (http://eumicrobedb.org) and EnsemblProtist (http://protists.ensembl.org) database were used for contig and EST information on *Hpa*. Several web servers, including InterPro (Quevillon et al., 2005) (http://www.ebi.ac.uk/interpro/), SMART (Letunic et al., 2012) (http://smart.embl-heidelberg.de/) and Pfam (Punta et al., 2012) (http://pfam.xfam.org) were used for the analysis of putative effector proteins. The *Hpa* genome (v8.3; http://eumicrobedb.org/genome/) was used to search for all possible ORFs, considering ATG as potential start codon. Web servers for SignalP V4.0 (Petersen et al., 2011) (http://www.cbs.dtu.dk/services/SignalP/) and TMHMM (Krogh et al., 2001) (http://www.cbs.dtu.dk/services/TMHMM/) were used to examine each ORFs for the presence of a signal peptide and transmembrane helices. Primer designs, *in silico* digests and comparison of genomic and full-length cDNA sequences of candidate genes were performed using Geneious (v8.0) (Kearse et al., 2012).

### Accession numbers

The raw sequence reads from the genomics sequencing of bulks are available from the Sequenced Read Archive (SRA) under accession numbers SRX1646609 (avirulent) and SRX1646555 (virulent). Genomic sequences of parental isolates can be found under accession numbers SRX1646645 for *Hpa*-Cala2 and SRX1646646 for *Hpa*-Noks1. Accession numbers for the *HAC1* and *S-HAC1* alleles are KX523274, KX523275, KX523276, KX523277, KX523278, KX523279, and KX523280. Genome sequence assemblies for the parental isolates are available from GenBank under accession numbers LKIA00000000.1 and LLKM00000000.1 (Studholme and Tör, to be published elsewhere). Accession number of *Hpa*-Noks1 23137 contig is LLKM01000918. Accession number for *Hpa*-Emoy2 reference genome v8.3 Scaffold 41 is ABWE00000000.2.

## Results

### Discovering cryptic avirulence determinant from mating of virulent *Hpa* isolates

**Figure 1:**
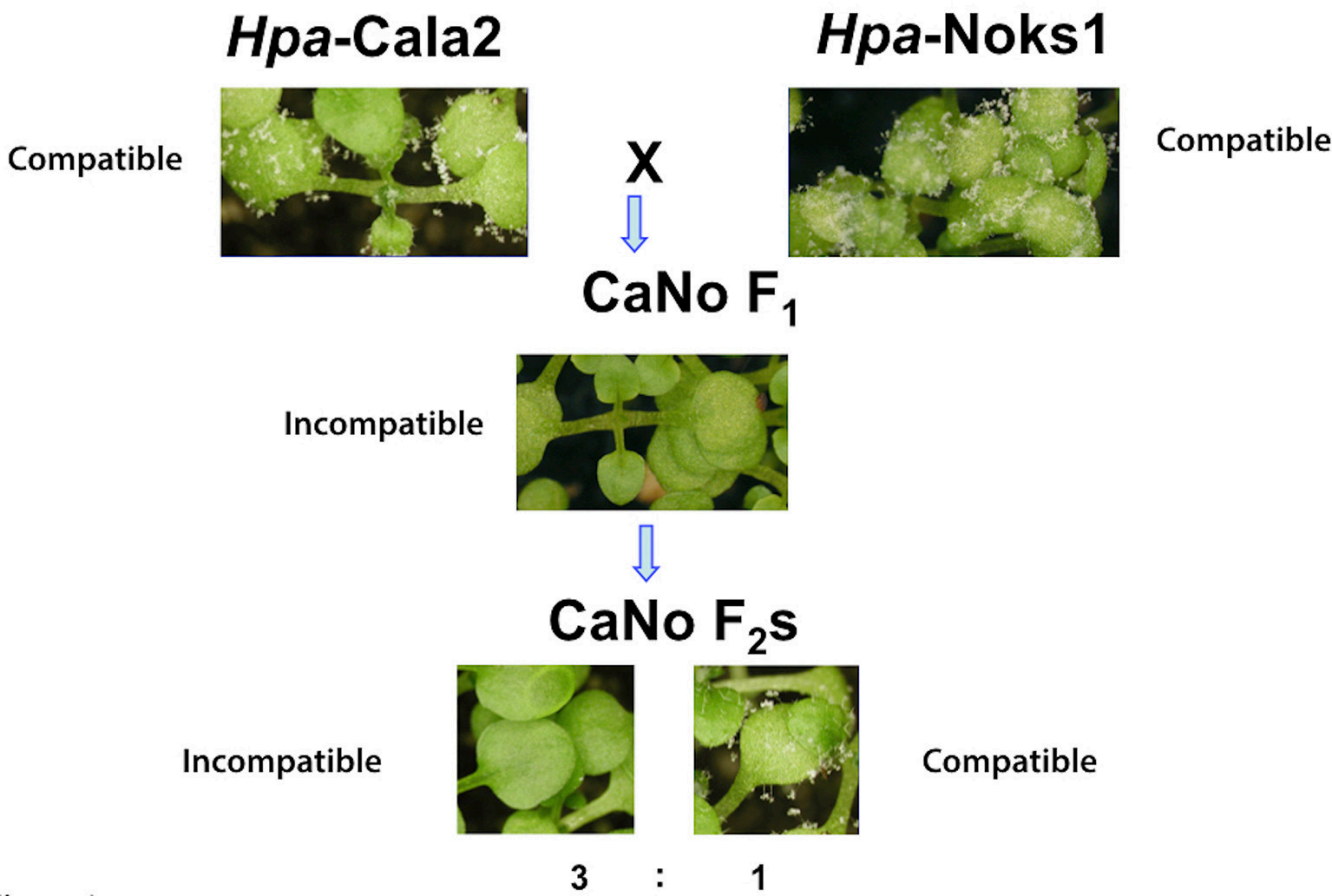
Avirulent determinant *HAC1* was identified from the segregating F_2_ population. The parental isolates *Hpa*-Noks1 and *Hpa*-Cala2 displayed a compatible interaction while CaNo F_1_ showed an incompatible interaction with RMX-A02 and CaNo F_2_ isolates segregating in a 3,1 (avirulent, virulent) ratio.

Two *Hpa* isolates, Cala2 and Noks1, were used to screen the worldwide diversity collection of 96 *A. thaliana* accessions (Nordborg et al., 2005) for variation in downy mildew response. Twenty-one accessions from this collection were susceptible in cotyledons to both of these isolates. The susceptible accessions were then screened with an F_1_ isolate (referred to hereafter as CaNo F_1_) that was generated from a laboratory mating of *Hpa*-Cala2 and *Hpa*-Noks1 (Bailey et al., 2011). Three North American accessions (RMX-A02, RMX-A180, and Yo-0) and one Swedish accession (Bil-7) were found to be resistant in cotyledons to CaNo F_1_ (Figure 1; Supplemental Table 2).

We used an available F_2_ mapping population from this F_1_ isolate for further investigations. Accessions that were susceptible to both *Hpa*-Cala2 and *Hpa*-Noks1 but resistant to CaNo F_1_ also showed resistance to some of the F_2_ isolates, indicating the presence of a possible “cryptic” avirulence locus in *Hpa*. Using the *A. thaliana* accession RMX-A02, a total of 54 randomly selected CaNo F_2_ isolates were screened to determine the inheritance of the avirulence determinant. A single dominant avirulence determinant could explain the phenotypic variation that segregated in the CaNo F_2_ population (avirulence, virulence ratio was 43,11, with chi-square = 0.717 and *P* = 0.30, Supplemental Table 3). The predicted avirulence determinant was designated as ***H****yaloperonospora* ***a****rabidopsidis* ***c****ryptic1* (*HAC1*). This result suggested the presence of a suppressor, designated *S**uppressor of* *HAC1* (*S-HAC1*) originating from either *Hpa*-Cala2 or *Hpa*-Noks1 but that was not inherited by the F_1_ isolate that was used to generate the F_2_ population.

### Fine mapping defines a 14 kb interval for the *HAC1* locus

**Figure 2:**
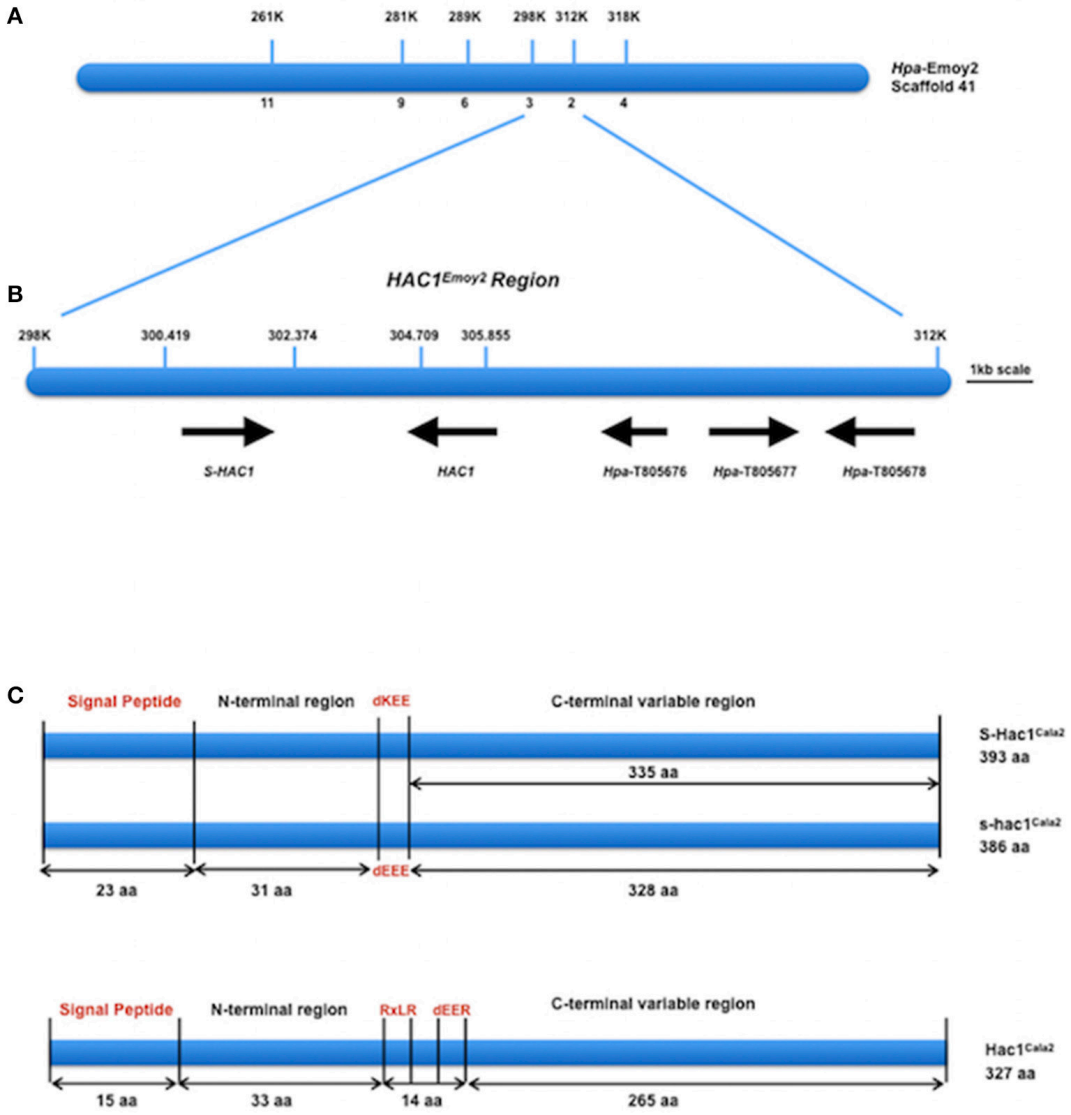
Map-based cloning of *HAC1*. **(A)** Position of molecular markers used to map the *HAC1* locus on the reference genome Emoy2 Scaffold 41. Numbers below the bar indicate the number of recombinants in 190 CaNo F_2_ individuals. **(B)** The genes where *HAC1* and *S-HAC1* are embedded in the locus. Predicted protein sequences of these genes suggest that they encode, *S-HAC1*, a secreted unknown protein and heterozygous in Cala2 genome; *HAC1*, a secreted unknown protein; *Hpa*-T805676, an unknown protein; *Hpa*-T805677, a NAD-dependant epimerase/dehydrase; *Hpa*-T805678, an actin-like protein. **(C)** Structures of S-HAC1^Cala2^, s-hac1^Cala2^ and HAC1^Cala2^. S-HAC1^Cala2^ has an initial signal peptide (1-23aa) and a dKEE motif (55-58aa). *s-hac1*^*Cala2*^ has an initial signal peptide (1-23aa) and a dEEE motif (55-58aa). Neither *S-HAC1*^*Cala2*^ nor *s-hac1*^*Cala2*^ had RxLR motifs. There were several polymorphic sequences between S-HAC1^Cala2^ and s-hac1^Cala2^. HAC1^Cala2^ has an initial signal peptide (1-15aa), an RxLR (49-52aa) and a dEER (59-62aa) motif. Signal peptide, dKEE/dEEE/dEER and RxLR are indicated in red.

DNA from 15 virulent and 15 avirulent F_2_ isolates was pooled in equal concentrations to make up the virulent and avirulent DNA bulks, respectively, and bulk segregant mapping analysis was performed. We generated 100 bp paired-end Illumina HiSeq2500 sequencing data from the two bulked (virulent and avirulent) pools, comprising 104 million reads for the virulent bulk, and 110 million reads for the avirulent bulk. We then used the *Hpa*-Noks1 genome assembly as a reference to map sequence reads from both virulent and avirulent bulks. A total of 26,722 SNP sites were then identified and examined for frequency of major alleles in the bulks. *Hpa*-Noks1 contig 23137 had the highest SNP frequency (Supplemental Table 4). Some of these SNPs were converted to CAPS marker and were then used to map the *HAC1* locus. Once linkage was confirmed, flanking markers were used to identify the *Hpa*-Emoy2 reference genome (v8.3.) Scaffold 41. A total of 190 F_2_ isolates were then screened with further markers and the *HAC1* locus was fine mapped to a 14 kb interval on the reference genome *Hpa*-Emoy2, Scaffold 41, 298.000-312.000 (Figure 2A).

### *HAC1^*Cala2*^* is an RXLR-dEER type avirulence determinant

According to the EnsemblProtists annotation, this 14 kb interval in *Hpa*-Emoy2 genome contains three genes without any putative effector domains and motifs, *Hpa*-T805676 encoding an unknown protein, *Hpa*-T805677 encoding a NAD-dependant epimerase/dehydrase (Pfam, PF01370), and *Hpa*-T805678 encoding an actin-like protein (PF00022). There were also two gaps in the *Hpa*-Emoy2 genome sequence assembly within the interval at position 41,300,419–302,374, and 41,304, 709–305,855 (Figure 2B). Subsequent efforts therefore focused on PCR amplification and sequencing to fill these gaps from genomic DNA of *Hpa*-Cala2, *Hpa*-Noks1, *Hpa*-Emoy2, and a virulent and an avirulent CaNo F_2_ isolates. This revealed the existence of a putative effector gene in each of the gaps. We then concentrated on these putative genes and a rapid isolation of cDNA ends (RACE) PCR was carried out for each. Full-length cDNAs for each gene from *Hpa*-Cala2, *Hpa*-Noks1, and *Hpa*-Emoy2 were obtained and comparison to genomic DNA revealed that none of them had any intron.

In the first gap (41,300,419–302,374), two alleles of the same gene with 96% identity at the nucleotide level were identified in *Hpa*-Cala2, indicating that this gene is heterozygous. Both alleles encode a predicted protein with structural similarity to other putative effector genes; a search for domains and motifs revealed a signal peptide and a dEEE motif in one allele (designated *s-hac1*^*Cala2*^, see below for details), and a signal peptide and a dKEE motif in the other (designated *S-HAC1*^*Cala2*^, see below for details). This indicates that both proteins may be secreted and act as putative effectors. Neither of the alleles had the RXLR motif (Figure 2C). Further analysis showed *Hpa*-Noks1 and *Hpa*-Emoy2 were both homozygous for the *s-hac1* allele. Additionally, the *Hpa*-Noks1 allele had a premature stop codon (see below for details).

In the second gap (41,304,709–305,855), a gene predicted to encode a protein with a signal peptide, an RxLR and a dEER motif was identified in *Hpa*-Cala2 (Figure 2C) suggesting that this protein may also be secreted and act as a putative effector. No heterozygosity was detected in this gene at *Hpa*-Cala2, *Hpa*-Noks1, and *Hpa*-Emoy2 genomes. Both *Hpa*-Noks1 and *Hpa*-Emoy2 alleles had premature stop codon.

*Hpa*-Noks1 and *Hpa*-Cala2 are virulent whereas CaNo F_1_ is avirulent in cotyledons of the accession RMX-A02. Since sequence information of the putative effector genes in the interval from *Hpa*-Noks1 and the virulent F_2_ isolates revealed that both have a premature stop codon, suggesting that both of them are non-functional. In contrast, *Hpa*-Cala2 and the avirulent homozygous F_2_ isolate have the full-length alleles indicating that the putative avirulence determinant may come from the parent isolate *Hpa*-Cala2.

**Figure 3:**
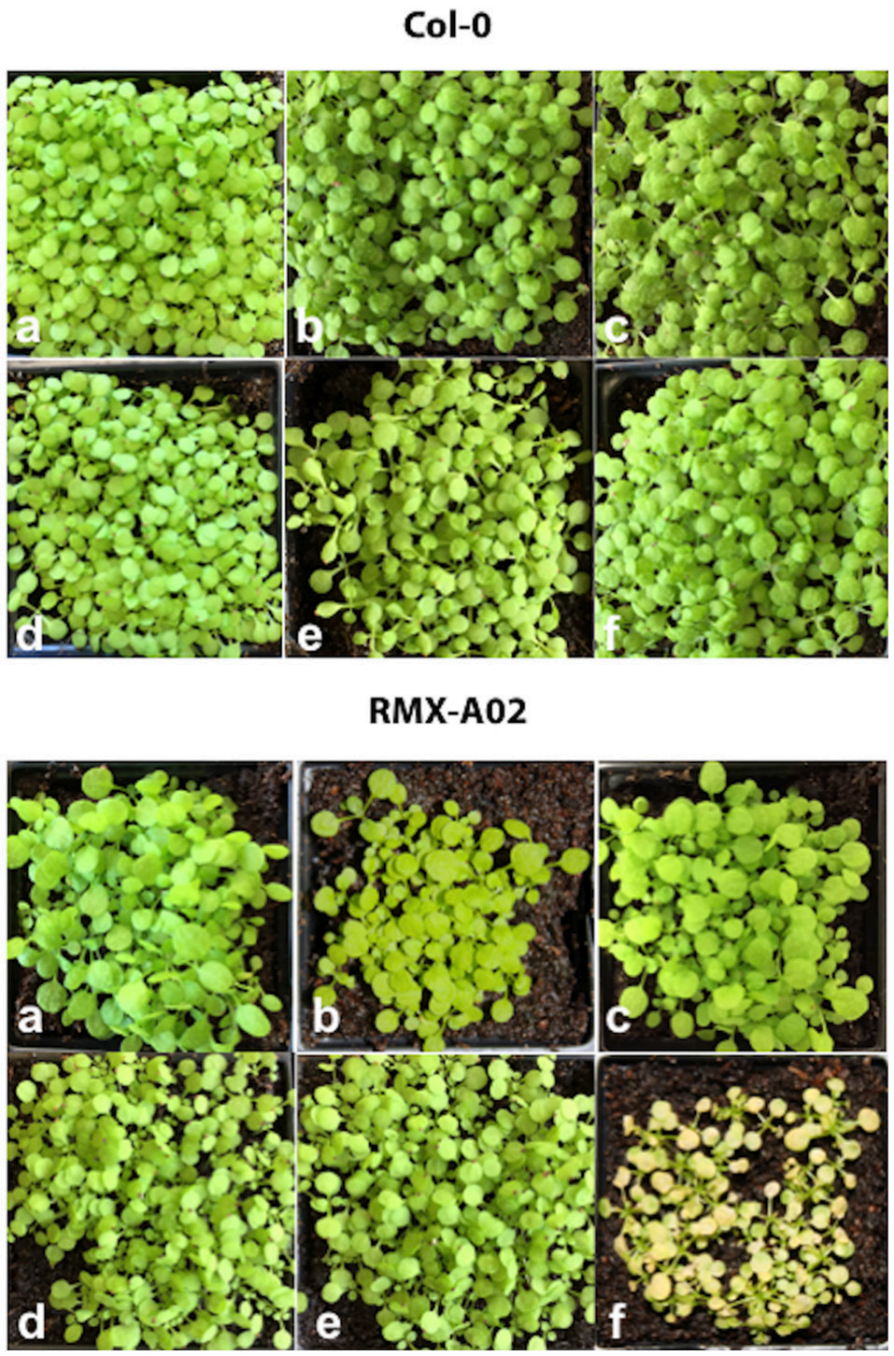
Activation of HAC1^Cala2^ induces cell death in transformed RMX-A02. Genes under the control of a dexamethasone (Dex)-activated promoter were transformed into *Arabidopsis* accessions Col-0 and RMX-A02 and T_3_ plants were treated with 30 μM Dex. **(a)**, non-transgenic, **(b)**, transgenic with *S-HAC1*^*Cala2*^, **(c)**, transgenic with *s-hac1*^*Cala2*^, **(d)**, transgenic with *s-hac1*^*Noks*1^, **(e)**, transgenic with *hac1*^*Noks*1^, **(f)**, transgenic with *HAC1*^*Cala2*^. Lines that carry *HAC1*^*Cala2*^ became chlorotic 3 days after treatment.

Recognition of the *HAC1* product by the corresponding *R*-gene in RMX-A02 protein should initiate a defense response and therefore, be amenable to identify the avirulent determinant. To test this, we cloned the full-length copies of the putative effector genes (with dEEE or DKEE motif in the first gap, and with RXLR/dEER in the second gap) from *Hpa*-Cala2 and *Hpa*-Noks1 into vectors with a Dex-inducible promoter and then transformed them into RMX-A02 and Col-0 plants. Transgenic T_1_, T_2_ and homozygous T_3_ plants were selected using Basta and the insert was confirmed with PCR (Supplemental Figure 1). All of the Basta-selected lines were treated with dexamethasone. Only the RMX-A02 seedlings that carried the RXLR-type effector from *Hpa*-Cala2 showed a chlorotic response 3 days after treatment (Figure 3) indicating an *R*-gene mediated cell death. Growth of these plants was stunted and in many cases individuals could not be recovered for producing seed. No altered phenotype was observed in any of the transgenic Col-0 plants indicating the defense response was race specific. Similarly, neither the *Hpa-Noks1* variant of the RXLR-type effector nor the variant of the second dEEE/dKEE type putative effectors triggered a defense response in RMX-A02 (Figure 3). This supports the conclusion that *HAC1* avirulence in *A. thaliana* accession RMX-A02 is determined by recognition of the RxLR-type protein inherited from *Hpa*-Cala2 and this *Avr* gene is designated *HAC1*^*Cala2*^.

### Pathogen genetics supports heterozygosity in *HAC1* locus in *Hpa*-Cala2

**Figure 4:**
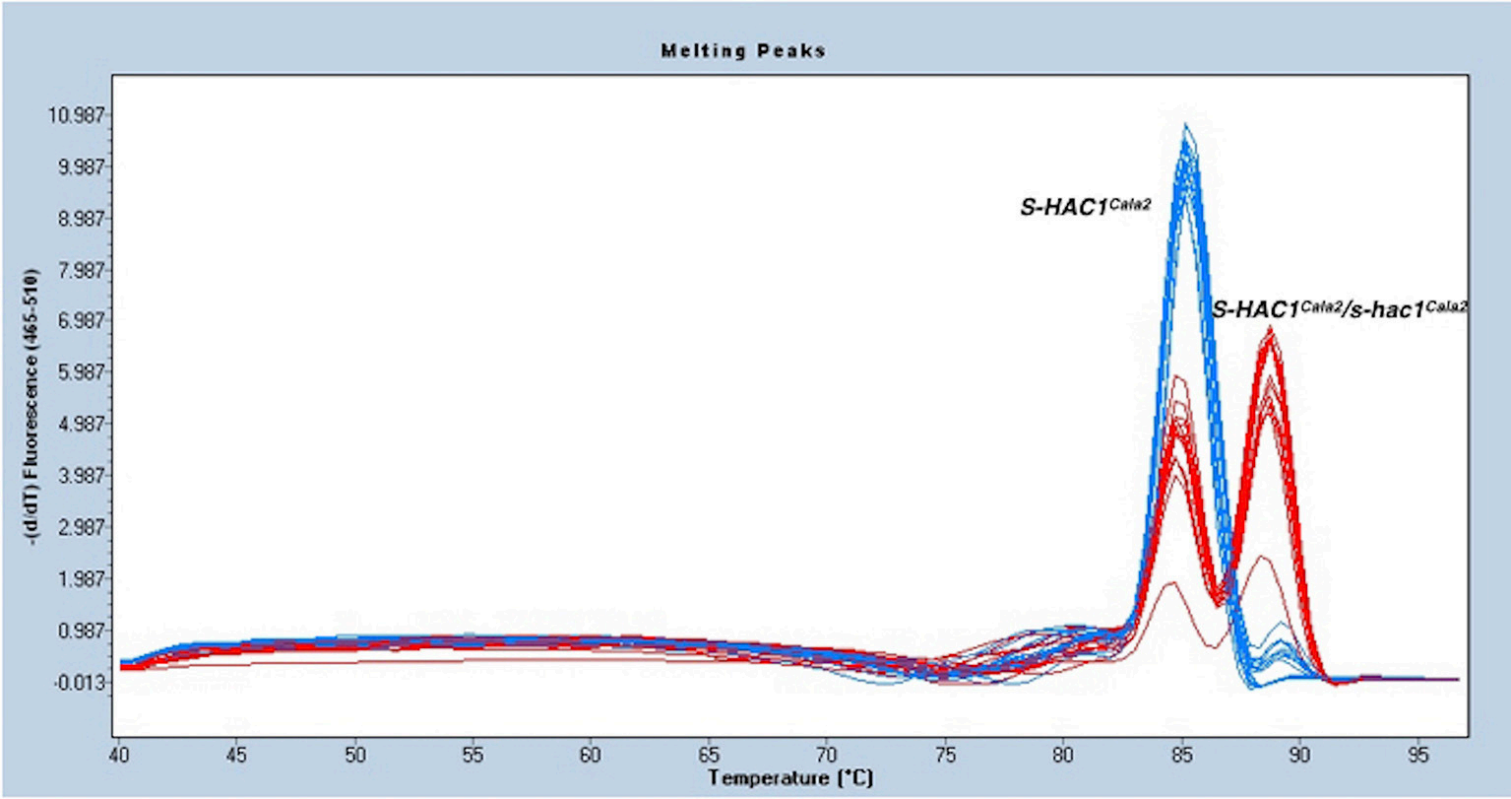
*S-HAC1*^*Cala2*^ *and s-hac1*^*Cala2*^ *alleles segregate in selfed Hpa*-Cala2. *Twenty-nine* cultures derived from oospores of selfed *Hpa*-Cala2 were subjected to high-resolution melt curve analysis using *S-HAC1*^*Cala2*^-specific primers. Blue peaks show homozygous *S-HAC1*^*Cala2*^ lines and red peaks show heterozygous lines. The experiment has been repeated with the *s-hac1*^*Cala2*^ specific primers and the same results were obtained.

As described above, the gene discovered in the first gap of the genome sequence of *Hpa*-Cala2 is heterozygous, with alleles having either a dEEE or dKEE motif. Both virulent and avirulent CaNo F_2_ isolates had only the putative effector with the dEEE motif, indicating that this allele does not influence the virulence of the pathogen on RMX-A02. This implies that the dEEE allele rather than the dKEE allele from *Hpa*-Cala2 was inherited during the generation of the F_1_. Thus, the presence of the dKEE allele in *Hpa*-Cala2 may somehow suppress the HAC1^Cala2^-triggered defense response. If true, then cultures derived from selfed-oospores of *Hpa*-Cala2 should segregate for phenotypic variation of virulence/avirulence in RMX-A02. To test this, 29 selfed *Hpa*-Cala2 lines were screened using high-resolution melt curve (HRM) analysis. The segregation ratio obtained was 8,15,6 ratio (dKEE/dKEE, dKEE/dEEE, dEEE/dEEE; 1,2,1 ratio) confirming the segregation of alleles (Figure 4). Seven of these selfed lines were used to test interactions on RMX-A02. Five lines were virulent and two avirulent correlating well with the HRM data. These results indicate that the dKEE allele is epistatic over *HAC1*^*Cala2*^ either by inducing susceptibility before HAC1^Cala2^ triggers defense or by somehow interfering directly with the function of the HAC1^Cala2^ effector. Since *Hpa*-Cala2 is virulent and heterozygous, the dKEE-containing allele is the originally predicted dominant allele, designated *S*-*HAC1*^*Cala2*^ (*S**uppressor of* *H*. *a**rabidopsidis* *c**ryptic 1*), whereas the allele with the dEEE motif would be the recessive or *s-hac1*^*Cala2*^ allele.

### Overexpression of *S-HAC1^*Cala2*^* in *Arabidopsis* alters interaction phenotype

Since pathogen genetics and molecular studies suggested that S-HAC1^Cala2^ plays a role in *Hpa*-Cala2 virulence on RMX-A02, we tested whether overexpression of this gene within the plant alters the *HAC1*^*Cala2*^-triggered defense response. The *S-HAC1*^*Cala2*^ gene was cloned under a 35S promoter, transformed into RMX-A02 plants and inserts were confirmed by PCR (Supplemental Figure 2). Homozygous T_3_ lines were then challenged with a representative avirulent F_2_ isolate (CaNo F_2_ 110) and *Hpa*-Cala2 and *Hpa*-Emoy2 as virulent control isolates. In controls, transgenic lines inoculated with *Hpa*-Cala2 or *Hpa*-Emoy2 did not show any alterations in pathogen growth and the lines gave wild-type levels of susceptibility.

**Figure 5:**
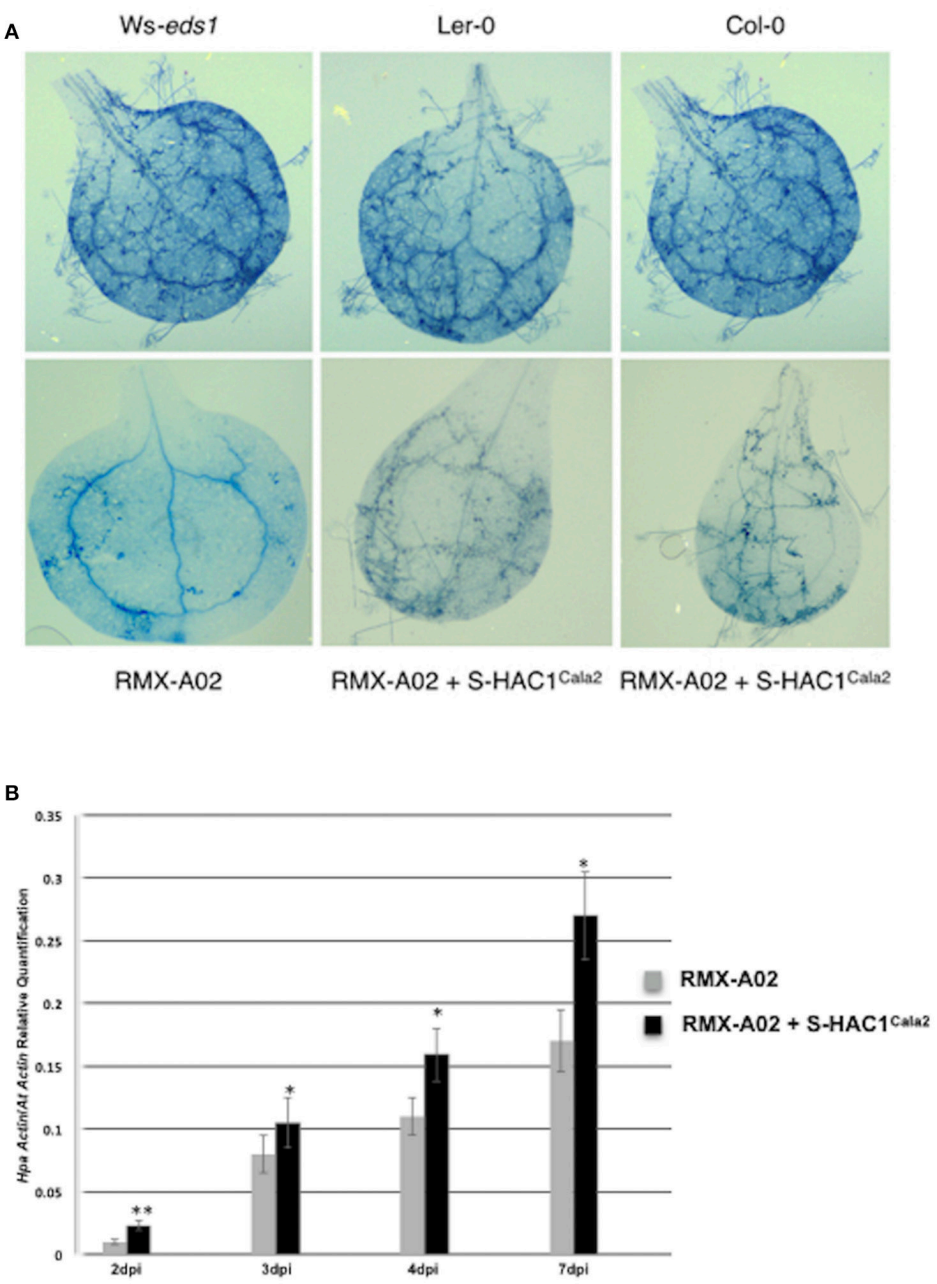
Overexpression of *S*-*HAC1*^*Cala2*^ in RMX-A02 displays altered phenotype and biomass production. The *S-HAC1*^*Cala2*^ gene was cloned under a 35S promoter and introduced to RMX-A02 plants. Homozygous T_3_ lines were challenged with avirulent F_2_ isolate, CaNo F_2_ 110. **(A)**, wild-type and transgenic seedlings inoculated with CaNo F_2_ 110 and stained with trypan blue 7 days post inoculation (dpi); **(B)**, pathogen biomass production in transgenic and non-transgenic RMX-A02. Relative abundance of *Hpa-Actin* (*Hpa-807716*) to *Arabidopsis At5g46630* gene was calculated from infected plant materials 2, 3, 4, and 7 dpi. Three samples were included for each analysis and the experiments were repeated three times. The increase in pathogen biomass in RMX-AO2+*S-HAC1*^*Cala2*^ compared with that in the RMX-A02 control was statistically significant (^*^P < 0.05, ^**^P < 0.01; Student's *t*-test).

The typical defense response of untransformed RMX-A02 seedlings inoculated with CaNo F_2_ 110 was without any symptoms such as flecks, pitting, or trailing necrosis; the cotyledons remained green and very rarely sporulation was observed. RMX-A02 lines carrying the *S-HAC1*^*Cala2*^ gene exhibited wild-type development of rosettes and flowering, indicating that overexpression of this gene did not have a physiological effect on the plant. However, T_3_ transgenic lines challenged with avirulent CaNo F_2_ 110 displayed enhanced sporulation, at L3 level interaction phenotype (Tör et al., 2002). Enhanced pathogen growth was also confirmed with trypan blue staining of infected tissues (Figure 5A). High-level sporulation expected for full susceptibility was not observed, indicating the transgene did not fully suppress the avirulence conferred by HAC1^Cala2^ when expressed *in planta*. Ws-*eds1*, Ler-0, and Col-0 inoculated with CaNo F_2_ 110 displayed full sporulation as expected (Figure 5A).

The biomass of CaNo F_2_ 110 within the transgenic and non-transgenic lines was also quantified using qPCR. A significant increase in pathogen biomass was detected within the RMX-A02 lines carrying the *S-HAC1*^*Cala2*^ gene lines (Figure 5B) indicating the presence of the *S-HAC1*^*Cala2*^ gene contributes to pathogen growth.

### Sequence and expression analysis of *HAC1^*Cala2*^, S-HAC1^*Cala2*^* and *s-hac1^*Cala2*^*

**Figure 6:**
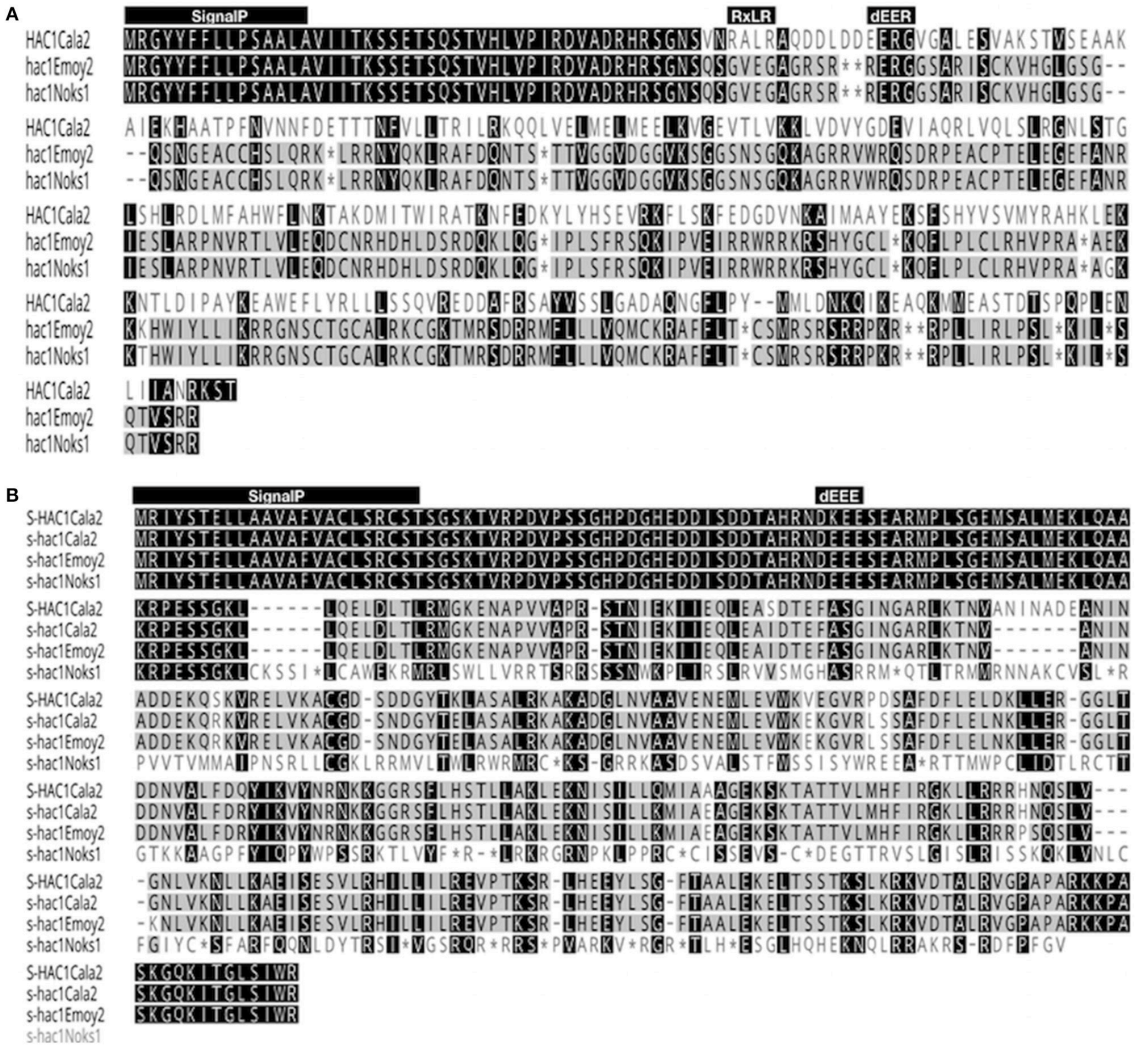
Alignment of amino acid sequences of HAC1 and S-HAC1. Amino acid sequences of HAC1 and S-HAC1 were predicted from cDNAs isolated from *Hpa*-Cala2, *Hpa*-Noks1 and *Hpa*-Emoy2 and were aligned using Geneious (v8.0). **(A)** Alignment of HAC1^Cala2^, HAC1^Noks1^, and HAC1^Emoy2^ amino acid sequences. Black or dark gray boxes with white letters indicate identity or similarity, respectively, to HAC1^Cala2^ and dashes indicate gaps in the alignment. The reading frames are adjusted to fit translation of regions downstream of stop codon. The predicted N-terminal signal peptide, the RXLR and dEER motifs are shown above the sequences, **(B)** Alignment of amino acid sequences of S-HAC1^Cala2^, s-hac1^Cala2^, s-hac1^Noks1^ and s-hac1^Emoy2^. Black or dark gray boxes with white letters indicate identity or similarity, respectively, to S-HAC1^Cala2^ and dashes indicate gaps in the alignment. The predicted N-terminal signal peptide and the dEER motifs are shown above the sequences. ^*^indicates stop codon.

The open reading frame of the *HAC1*^*Cala2*^ gene encodes a predicted protein of 327 amino acids (molecular mass of 37.262 kDa). Domain and motif searches of the HAC1^Cala2^ protein identified a signal peptide (M1-A15), an RxLR (49 to 52 aa) and dEER (59-62 aa) motifs (Figure 6A). Transmembrane prediction using the TMHMM server (Krogh et al., 2001) did not identify any TM domain, suggesting that HAC1^Cala2^ is a soluble protein. Analysis of HAC1^Noks1^ and HAC1^Emoy2^ sequences indicated that these proteins are identical at the N-terminal, but both of them lack the RXLR motif and both have a premature stop codon around the dEER (58 aa) motif (Figure 6A). This stop codon has originated from indels at the nucleotide level that cause a frame shift (Supplemental Figure 3). BLAST searches against the *Hpa*-Emoy2 database (http://eumicrobedb.org) did not reveal a significant alignment of HAC1^Cala2^ with any unigene. Using HAC1^Cala2^, the TBLASTN search against Ensembl EST database revealed 66% identity to HpaT801867 (ATR1^NDWSB^ allele) and 48% identity to HpaT813746, two avirulence protein-like proteins. Analysis of structure of the HAC1^Cala2^ protein using Phyre2 (Kelley et al., 2015) revealed that 55% of the total amino acid sequences can be modeled onto the ATR1 effector protein from *Hpa*, supporting the hypothesis that the HAC1^Cala2^ protein may act as an effector protein (Supplemental Figure 4).

The open reading frame of the *S-HAC1*^*Cala2*^ allele encodes a predicted protein of 393 aa (molecular mass of 43.225 kDa). Domain and motif searches on the S-HAC1^Cala2^ protein identified a signal peptide (M1-T23), and a dKEE (55-58 aa) motif (Figure 6B), and no TM domain has been identified. s-*hac1*^*Cala2*^ has similar motifs but has the dEEE instead of dKEE (Figure 6B). S-HAC1^Cala2^ and s-hac1^Cala2^ share 94.9% identity. s-hac^Noks1^ has a premature stop codon (94 aa) and analysis of S-HAC1^Cala2^, s-hac1^Cala2^, s-hac^Noks1^, and s-hac^Emoy2^ sequences revealed that the N-termini of these proteins are conserved (Figure 6B). Alignment of nucleotide sequences of S-HAC1^Cala2^, s-hac1^Cala2^, s-hac^Noks1^, and s-hac^Emoy2^ indicate the level of polymorphism (Supplemental Figure 5). BLAST and TBLASTN searches with S-HAC1^Cala2^, s-hac1^Cala2^ against Ensembl did not reveal any significant alignment. In addition, structure analysis of S-HAC1^Cala2^ and s-hac1^Cala2^ using Phyre2 did not detect any (remote) homologs for the protein.

**Figure 7:**
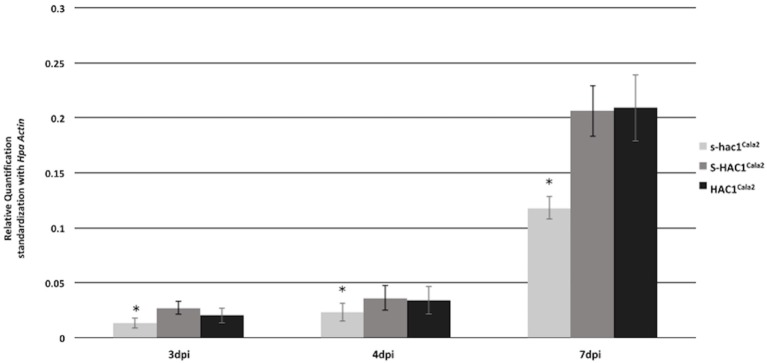
Expression analysis of *s-hac1*^*Cala2*^, *S-HAC1*^*Cala2*^ and *HAC1*^*Cala2*^. Total RNA was isolated from infected *Hpa*-Cala2 at 3, 4 and 7dpi. Quantitative Real-time PCR was carried out using gene specific primers for *HAC1*^*Cala2*^*, S-HAC1*^*Cala2*^ and *s-hac1*^*Cala2*^. Fifteen seedlings from each genotype made up a sample and three replicates were used for each sample, the experiments were repeated three times with similar results. Expression level of *s-hac1*^*Cala2*^ was significantly lower than that of *S-HAC1*^*Cala2*^ and *HAC1*^*Cala2*^ in *Hpa*-Cala2 background. Asterisks denote statistical significance in two-tailed Student's *t*-test (*p* < 0.05).

Expression of *HAC1*^*Cala2*^ *and S-HAC1*^*Cala2*^ in *Hpa*-Cala2 was investigated over a time course. Total RNA was isolated from uninfected (control) or infected tissues at 1–7 days post inoculation (dpi). Gene specific primers were used for reverse transcriptase mediated (RT) PCR analysis (Supplemental Figure 6). Expression of both genes was detected in infected tissues at 2 dpi. To obtain further insight into the expression level of these genes, quantitative PCR was carried out with total RNA isolated from *Arabidopsis* seedlings infected *Hpa*-Cala2 at 3, 4, and 7 dpi using gene specific primers for *HAC1*^*Cala2*^*, S-HAC1*^*Cala2*^ and *s-hac1*^*Cala2*^. All three genes showed increasing levels of expression over a week, and the expression levels of *HAC1*^*Cala2*^ and *S-HAC1*^*Cala2*^ were very similar. The expression level of *s-hac1*^*Cala2*^ was significantly lower than that of *HAC1*^*Cala2*^ and *S-HAC1*^*Cala2*^ (Figure 7).

### Other heterozygous suppressor genes exists in *Hpa*-Cala2

To investigate whether any other heterozygous alleles might exist, we used the parent isolate *Hpa*-Cala2 and two progeny lines from selfed *Hpa*-Cala2, *Hpa*-Cala2S2, and *Hpa*-Cala2S5, to screen 50 of the 96 worldwide diversity collection of *A. thaliana* accessions (Nordborg et al., 2005). Interestingly, the isolates displayed different interaction phenotypes with these accessions (Supplemental Table 5), indicating the parent isolate was heterozygous at other loci. Forty accessions gave the same reaction to *Hpa*-Cala2, *Hpa*-Cala2S2, and *Hpa*-Cala2S5. However, 10 of the accessions differed in their responses, including eight (Bil-7, Tamm-2, Rmx-A02, Eden-2, Mz-0, Kz-1, Rmx-A180, Ra-0) that were susceptible to the parent isolate *Hpa*-Cala2. Only four of these accessions (Mz-0, Kz-1, Sq-8, Ws-0) were susceptible to *Hpa*-Cala2S2, and only six (Bil-7, Tamm-2, Rmx-A02, Eden-2, Rmx-A180, Ra-0) were susceptible to *Hpa*-Cala2S5 (Table 1). These findings suggest that heterozygosity may exist and be maintained in other loci of the *Hpa*-Cala2 genome.

**Table 1:** Interaction phenotypes of *Arabidopsis* accessions with the parent isolate *Hpa*-Cala2 and two offspring.

**Accessions**	***Hpa*-Cala2**	*Hpa*-Cala2S2^*^	Hpa-Cala2S5^*^
Bil-7	S	R	S
Tamm-2	S	R	S
Rmx-A02	S	R	S
Eden-2	S	R	S
Mz-0	S	S	R
Kz-1	S	S	R
Rmx-A180	S	R	S
Sq-8	R	S	R
Ra-0	S	R	S
Ws-0	R	S	R

S, Susceptible; R, Resistance;

* *Hpa-Cala2S2 and Hpa-Cala2S5 are derived from oospores generated from Hpa-Cala2*.

## Discussion

Cryptic avirulence is a phenomenon that was first described by H.H. Flor during his seminal research on interactions between flax and the basidiomycete fungus *M. lini* (Flor, 1946) and this phenomenon has subsequently been confirmed in fungal genetic experiments by other researchers (Lawrence et al., 1981; Ellingboe, 1992; Lau et al., 1993). Here we present pathology, genetic and molecular evidence that this phenomenon also occurs in a biotrophic oomycete. Using the *Arabidopsis*-*Hyaloperonospora* experimental model, we showed that the *Arabidopsis* accession RMX-A02 is susceptible to two standard isolates (*Hpa*-Cala2 and *Hpa*-Noks1) but resistant to an F_1_ derived from these isolates (CaNo F_1_) and that a single corresponding avirulence locus is indicated by segregation in F_2_ progeny derived from this F_1_. This was confirmed using fine-mapping and genomic sequencing to clone an avirulence determinant (designated *HAC1*^*Cala2*^) and a heterozygous avirulence suppressor gene in the same map interval (designated *S-HAC1*^*Cala2*^*/s-hac1*^*Cala2*^). Both genes encode putative effector proteins.

In most cases, a map-based gene cloning strategy uses parental lines that differ in contrasting phenotypes (e.g., resistant vs. susceptible plants; or avirulent vs. virulent pathogen isolates) to generate a mapping population. However, the parent isolates used in the present study (*Hpa*-Cala2 and *Hpa*-Noks1) were both virulent on the same *Arabidopsis* accession, RMX-A02. Thus, we could not know from which parent the hidden or cryptic avirulence allele originated. Cryptic alleles are phenotypically silent DNA sequences and have been reported in prokaryotic microorganisms as well as in fungi (Hall et al., 1983; Le Gac et al., 2007). Several molecular explanations have been postulated including, (1) spontaneous occurrence by mutation or rearrangements, (2) action of a second suppressor locus, and (3) epigenetic control. They may however be activated in a few individuals of a large population by mutation, recombination, insertion elements or by other genetic mechanisms (Hall et al., 1983; Li, 1984).

In the present investigation, we used next generation sequencing to reveal the loci involved. Virulent and avirulent bulks were sequenced, as well as the genomes of the parental isolates, and we used the polymorphic nature of the isolates to screen 26722 SNP sites, enabling us to identify a contig from *Hpa*-Noks1 genome linked to the locus. The *HAC1* locus was mapped to a 14 kb interval using an experimental population of 190 F_2_ isolates, and haplotype sequence information from *Hpa*-Cala2, *Hpa*-Noks1, and *Hpa*-Emoy2 to characterize the high degree of polymorphism in the interval.

All of the *R/AVR* gene pairs that have previously been cloned from the *Hpa-Arabidopsis* system, including *RPP1/ATR1* (Botella et al., 1998; Rehmany et al., 2005) and *RPP13/ATR13* (Bittner-Eddy et al., 1999; Allen et al., 2004), have fallen into the NLR type of *R*-genes and RxLR-dEER class of effectors. HAC1^Cala2^ has the canonical signal and translocation peptides, RxLR-dEER, and triggers a defense response in RMX-A02. Previously, we used the CaNo F2 110 to investigate the corresponding gene in RMX-A02 and mapped the locus in the *Arabidopsis* genome to chromosome 4 within an interval of 35 kb containing three *R*-genes in the *RPP4/RPP5* locus (unpublished data). Thus, HAC1^Cala2^-triggered defense responses in the RMX-A02 background are very highly likely to be mediated by an NLR type *R*-gene.

A large number of RxLR or RxLR-like effectors have been identified in various downy mildew pathogens and *Phytophthora* species (Bailey et al., 2011; Anderson et al., 2015) and molecular studies on several of these effectors have shown that they suppress plant immunity (Sohn et al., 2007). It was interesting that the structure of HAC1^Cala2^ could be modeled with high confidence onto the ATR1 effector protein. To investigate the RPP1/ATR1 interaction, Chou et al. (2011) determined the crystal structure of ATR1 and identified the critical recognition sites of the effector protein. They concluded that oomycete effectors that share the same lineage as ATR1 rapidly evolve to escape host detection. In this case, if HAC1^Cala2^ is indeed from the same family as ATR1, it can be proposed that *HAC1*^*Cala2*^ alleles, *hac1*^*Noks*1^, and *hac1*^*Emoy*2^, have already evolved to avoid recognition as they have premature stop codons, enabling *Hpa*-Noks1 and *Hpa*-Emoy2 to be virulent on RMX-A02.

A diploid organism can be heterozygous at a gene locus when its cells contain two different alleles of a gene and use this for its own advantage. The importance of heterozygosity for virulence in fungi and oomycetes has been recognized for a while and in some cases documented. For example, a high level of heterozygosity for virulence in two populations of *Puccinia recondita* (wheat leaf rust fungus) has been found (Kolmer, 1992). Similarly, genomic sequences of *P. striiformis* f. sp. *tritici* (*Pst*- wheat stripe rust fungus) revealed the presence of heterozygosity (Zheng et al., 2013). Population studies on *M. grisea* (rice blast fungus) using PCR markers identified six of the loci investigated to be heterozygous (Babu et al., 2013). Genomic investigations on *Phytophthora capsici* revealed the loss of heterozygosity, which has been proposed to be a rapid mechanism for fixing alleles and may be an important component of adaptability (Lamour et al., 2012). Similarly, screening of 652 *P. infestans* isolates from commercial potato fields in the Netherlands during a ten-year period using 12 informative microsatellite markers and mitochondrial haplotypes detected a low level of heterozygosity (Li et al., 2012). It has been reasoned that a pathogen could gain a selective advantage from a heterozygous genotype by increasing the pathogen's ability to adapt to a changing host environment such as overcoming the host's resistance mechanism (Zheng et al., 2013).

**Figure 8:**
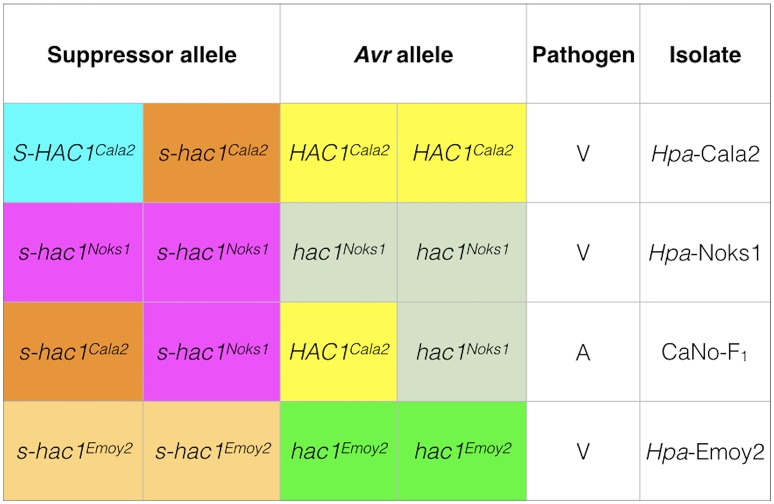
Genotypes of three *Hpa* isolates at the *HAC1* locus. Sequencing and pathology results were used to determine the genotypes of isolates. In *Hpa*-Cala2, virulence on RMX-A02 results from the combination of an active suppressor (*S-HAC1*^*Cala2*^) and an active avirulence (*HAC1*^*Cala2*^) allele. In the presence of an inactive suppressor (*s-hac1*^*Cala2*^) and an active avirulence (*HAC1*^*Cala2*^) allele, the interaction results in avirulence. In *Hpa*-Noks1 and *Hpa*-Emoy2, the putative avirulence gene is inactive due to mutations, thus both are virulent on RMX-A02.

In the current investigation, it is interesting that heterozygosity of a putative effector gene was revealed in one isolate *Hpa*-Cala2 but not in two other standard isolates (*Hpa*-Noks1 and *Hpa*-Emoy2) (Figure 8). Heterozygote genotypes are known to have a higher relative fitness than either the homozygote dominant or homozygote recessive genotypes, known as heterozygous advantage (Hedrick, 2012). There are many examples of heterozygous advantage in living systems. For example, the Major Histocompatibility Complex (MHC) loci are highly polymorphic heterozygous loci that control immunological recognition of pathogens in animals and confer a selective advantage by enhancing resistance to infectious diseases (Penn et al., 2002). A well-established case of heterozygous advantage in humans is that of the genes involved in sickle cell anemia (Luzzatto, 2012) and Cystic Fibrosis (Modiano et al., 2007). In plant breeding programmes, hybrid vigor generated from crosses between inbred lines from different genetic backgrounds, is an example of heterozygous advantage used routinely for higher crop yield (Whitford et al., 2013). However, the majority of studies on heterozygosity in fungi and oomycete pathogens have been within the field of population genetics and there has been no investigation of heterozygosity at the level of individual locus or effector genes. Segregation of a suppressor/effector after sexual reproduction (as observed in this study) means that a proportion of the progeny will have an active Avr effector, whereas others will not. This may be advantageous to the pathogen by increasing the pathogen's ability to adapt to a changing host environment.

A secreted effector can both trigger and suppress *R* gene-based immunity. For example, *F. oxysporum* f.sp. *lycopersici* (*Fol*) effector *Avr1* triggers a defense response when the host plant, tomato, carries a matching *R*-gene (*I* or *I-1*). However, Avr1 suppresses the protective effect of two other host *R*-genes, *I-2* and *I-3* (Houterman et al., 2008). Similarly, IPI-04, an effector protein of *P. infestans*, has been shown not only to elude detection by the host potato protein RB, but also to block recognition of the effector protein IPI-01, leading to suppression of RB-mediated host cell death (Halterman et al., 2010; Chen et al., 2012). A similar scenario may exist for the *Hpa*-Cala2 RMX-A02 interactions, where S-HAC1^Cala2^ may suppress the HAC1^Cala2^-triggered host immunity or interfere with its function. Suppression of HAC1^Cala2^-triggered immunity may occur within the host cell. For this to happen, S-HAC1^Cala2^ has to be delivered inside the host cell and may target the same host component as HAC1^Cala2^. Alternatively, S-HAC1^Cala2^ may physically bind to HAC1^Cala2^ within the pathogen or inside the host cell, promoting conformational changes within HAC1^Cala2^ and preventing either the secretion of HAC1^Cala2^ from the pathogen, its entry into the host cell or the recognition of HAC1^Cala2^ by the host intracellular receptor. We hope to investigate this further with yeast two-hybrid and pull-down experiments.

Overexpression of *S-HAC1*^*Cala2*^ in RMX-A02 plants did not confer any resistance phenotype. However, when these transgenic lines were challenged with the avirulent CaNo F_2_ 110 isolate (carrying *HAC1*^*Cala2*^ and *s-hac1*^*Cala2*^), phenotypic alterations were observed, indicating that S-HAC1^Cala2^ may have interacted with a pathogen-delivered protein, most likely to be HAC1^Cala2^, in the apoplast or cytoplasm of the host cell. Surprisingly, full suppression of HAC1^Cala2^–triggered resistance was not observed in these transgenic lines carrying *S-HAC1*^*Cala2*^. This may indicate that S-HAC1^Cala2^ needs to interact with HAC1^Cala2^ within the pathogen or apoplast. Alternatively, it may require posttranslational modifications (PTM) by the pathogen, such as phosphorylation, SUMOylation, methylation, ubiquitination, or acetylation (Hewezi, 2015). Nevertheless, the results of this study suggest that the combination of HAC1^Cala2^ and S-HAC1^Cala2^ may dictate the race-specificity of *Hpa*-Cala2 on the *Arabidopsis* accession RMX-A02.

*Hpa* is a diploid, homothallic pathogen, which means that a haploid antheridium and oogonium are produced within the same thallus to generate oospores. Frequent sexual reproduction in a homothallic pathogen would be expected to give rise to a largely homozygous genome and so maintenance of heterozygosity at the *HAC1* or other loci in *Hpa*-Cala2 may be surprising. However, this and many other experimental isolates have been originally single spored and maintained in the laboratory largely through asexual propagation. In a diploid clonal population, mutation events at each locus lead to an accumulation of heterozygosity over time (Balloux et al., 2003); thus, it would be expected that there may be considerable heterozygosity in experimental isolates where there is an absence of frequent sexual reproduction. In addition, sexual crosses between expressed and silenced *Avr* alleles can result in varying outcomes and unusual inheritance patterns for *Avr* gene expression in progeny (Na and Gijzen, 2016).

The use of selfed lines *Hpa*-Cala2S2 and *Hpa*-Cala2S5 indicated that other suppressor loci within *Hpa*-Cala2 might be heterozygous and so the question arises as to whether heterozygosity is a widespread phenomenon in *Hpa*. To investigate this, we took advantage of the fact that *Hpa* isolates have been used in different laboratories to screen the same *Arabidopsis* diversity collections and some published data are available. For example, in investigations by both Krasileva et al. (2011) and Nemri et al. (2010), interaction phenotypes of isolates *Hpa*-Emwa1 and *Hpa*-Emco5 (among other isolates) on the 83 Arabidopsis accessions were assessed. However, different results were obtained by these authors for at least 12 *Arabidopsis* accessions (Supplemental Table 6). This discrepancy may have arisen if each group obtained their isolates from different oospores of a segregating population, indicating the existence of heterozygosity at different loci. In addition, it should be remembered that natural populations of *Hpa* are polycyclic, reproducing many times asexually (leading to an increase in heterozygosity) as well as sexually, before overwintering as oospores; also, despite being homothallic, in mixed populations, outcrossing with other races is possible. Thus, it is very probable that natural populations of *Hpa* have considerable heterozygosity. Evidence of outcrossing in other homothallic oomycetes, such as *P. ultimum* (Francis and St. Clair, 1993) and *P. aphanidermatum* (Lee et al., 2010) has also been used to explain high levels of heterozygosity in these populations.

## Author contributions

MT and AW-T: Planned and designed the research; AW-T, MT, VC;, and OT: Conducted the laboratory work; AW-T, MT, and DS: Analyzed the data; MT, AW-T, EH, and DS: Interpreted the data and wrote the manuscript.

### Conflict of interest statement

The authors declare that the research was conducted in the absence of any commercial or financial relationships that could be construed as a potential conflict of interest.

## Supplementary material

Supplemental Figure 1 PCR confirmation of *HAC1*^*Cala2*^ inserts in transgenic RMX-A02 plants.

Supplemental Figure 2 PCR confirmation of inserts carrying *S-HAC1*^*Cala2*^ gene in the transgenic RMX-A02 plants.

Supplemental Figure 3 Alignment of nucleotide sequences of *HAC1*^*Cala2*^ and homologs from *Hpa*-Emoy2 and *Hpa*-Noks1.

Supplemental Figure 4 Amino acid sequences of HAC1^Cala2^ modeled onto the ATR1 effector 2 protein from *Hpa* using Phyr2.

Supplemental Figure 5 Alignment of nucleotide sequences of *S-HAC1*^*Cala2*^ homologs from *Hpa*-Cala2, *Hpa*-Emoy2, and *Hpa*-Noks1.

Supplemental Figure 6 Expression analysis of *HAC1*^*Cala2*^ *and S-HAC1*^*Cala2*^.

Supplementary Table 1 Primer sequences used in this study.

Supplementary Table 2 Screening Nordborg/Bergelson (NB) worldwide diversity collection of 96 *Arabidopsis* accessions with *Hpa*-Noks1, *Hpa*-Cala2, CaNo F2 110 and CaNo F_1_.

Supplementary Table 3 Interaction phenotypes (IP) recorded seven days after inoculation of RMX-A02 accession with CaNo F_2_ isolates.

Supplementary Table 4 Frequency of major alleles at SNP sites from different scaffolds and contigs of *Hpa*-Noks1 genome.

Supplementary Table 5 Interaction phenotypes of the parent isolate *Hpa*-Cala2 and two offspring with *Arabidopsis* accessions.

Supplementary Table 6 Comparison of interaction phenotypes obtained from two independent published studies of *Hyaloperonospora arabidopsidis* isolates *Hpa*-Emwa1 and *Hpa*-Emco5 on 83 *Arabidopsis* accessions of the Nordborg/Bergelson collection.

## References

[B1] Allen R. L. Bittner-Eddy P. D. Grenville-Briggs L. J. Meitz J. C. Rehmany A. P. Rose L. E.. (2004). Host-parasite coevolutionary conflict between Arabidopsis and downy mildew. Science 306, 1957–1960. 10.1126/science.1104022 15591208

[B2] Anderson R. G. Deb D. Fedkenheuer K. McDowell J. M. (2015). Recent progress in RXLR effector research. Mol. Plant Microbe Interact. 28, 1063–1072. 10.1094/MPMI-01-15-0022-CR 26125490

[B3] Babu T. K. Sharma R. Upadhyaya H. D. Reddy P. N. Deshpande S. P. Senthilvel S. (2013). Evaluation of genetic diversity in *Magnaporthe grisea* populations adapted to finger millet using simple sequence repeats (SSRs) markers. Physiol. Mol. Plant Pathol. 84, 10–18. 10.1016/j.pmpp.2013.06.001

[B4] Bailey K. Cevik V. Holton N. Byrne-Richardson J. Sohn K. H. Coates M.. (2011). Molecular cloning of ATR5 Emoy2 from *Hyaloperonospora arabidopsidis*, an avirulence determinant that triggers RPP5-mediated defense in Arabidopsis. Mol. Plant Microbe Interact. 24, 827–838. 10.1094/MPMI-12-10-0278 21361788

[B5] Balloux F. Lehmann L. de Meeûs T. (2003). The population genetics of clonal and partially clonal diploids. Genetics 164, 1635–1644. 12930767 10.1093/genetics/164.4.1635 PMC1462666

[B6] Baxter L. Tripathy S. Ishaque N. Boot N. Cabral A. Kemen E.. (2010). Signatures of adaptation to obligate biotrophy in the *Hyaloperonospora arabidopsidis* genome. Science 330, 1549–1551. 10.1126/science.1195203 21148394 PMC3971456

[B7] Bittner-Eddy P. Can C. Gunn N. Pinel M. Tör M. Crute I.. (1999). Genetic and physical mapping of the RPP13 locus in Arabidopsis responsible for specific recognition of several *Peronospora parasitica* (downy mildew) isolates. Mol. Plant Microbe Interact. 12, 792–802. 10.1094/MPMI.1999.12.9.792 10494631

[B8] Botella M. A. Parker J. E. Frost L. N. Bittner-Eddy P. D. Beynon J. L. Daniels M. J.. (1998). Three genes of the Arabidopsis RPP1 complex resistance locus recognize distinct *Peronospora parasitica* avirulence determinants. Plant Cell 10, 1847–1860. 10.1105/tpc.10.11.1847 9811793 PMC143951

[B9] Bourras S. McNally K. E. Müller M. C. Wicker T. Keller B. (2016). Avirulence genes in cereal powdery mildews, the gene-for-gene hypothesis 2.0. Front. Plant Sci. 7:241. 10.3389/fpls.2016.00241 26973683 PMC4771761

[B10] Chen Y. Liu Z. Halterman D. A. (2012). Molecular determinants of resistance activation and suppression by *Phytophthora infestans* effector IPI-0. PLoS Pathog. 8:e1002595 10.1371/annotation/75775518-f06e-4148-a639-31cfc6972b2e 22438813 PMC3305431

[B11] Chou S. Krasileva K. V. Holton J. M. Steinbrenner A. D. Alber T. Staskawicz B. J. (2011). Hyaloperonospora arabidopsidis ATR1 effector is a repeat protein with distributed recognition surfaces. Proc. Natl. Acad. Sci. U.S.A. 108, 13323–13328. 10.1073/pnas.1109791108 21788488 PMC3156156

[B12] Clough S. J. Bent A. F. (1998). Floral dip, a simplified method for Agrobacterium-mediated transformation of *Arabidopsis thaliana*. Plant J. 16, 735–743. 10.1046/j.1365-313x.1998.00343.x 10069079

[B13] Dodds P. N. Rathjen J. P. (2010). Plant immunity, towards an integrated view of plant–pathogen interactions. Nat. Rev. Genet. 11, 539–548. 10.1038/nrg2812 20585331

[B14] Doehlemann G. Hemetsberger C. (2013). Apoplastic immunity and its suppression by filamentous plant pathogens. New Phytol. 198, 1001–1016. 10.1111/nph.12277 23594392

[B15] Doyle J. J. (1987). A rapid DNA isolation procedure for small quantities of fresh leaf tissue. Phytochem. Bull. 19, 11–15.

[B16] Earley K. W. Haag J. R. Pontes O. Opper K. Juehne T. Song K.. (2006). Gateway-compatible vectors for plant functional genomics and proteomics. Plant J. 45, 616–629. 10.1111/j.1365-313X.2005.02617.x 16441352

[B17] Ellingboe A. H. (1992). Segregation of avirulence/virulence on three rice cultivars in 16 crosses of *Magnaporthe grisea*. Phytopathology 82, 597–601. 10.1094/Phyto-82-597

[B18] Eulgem T. Tsuchiya T. Wang X. J. Beasley B. Cuzick A. Tör M.. (2007). EDM2 is required for RPP7-dependent disease resistance in Arabidopsis and affects RPP7 transcript levels. Plant J. 49, 829–839. 10.1111/j.1365-313X.2006.02999.x 17253987

[B19] Flor H. H. (1946). Genetics of pathogenicity in *Melampsora lini*. J. Agric. Res. 73, 335–357.

[B20] Francis D. M. St. Clair D. A. (1993). Outcrossing in the homothallic oomycete *Pythium ultimum* detected with molecular markers. Curr. Genet. 24, 100–106. 10.1007/BF00324672 8102945

[B21] Göker M. Riethmüller A. Voglmayr H. Weiss M. Oberwinkler F. (2004). Phylogeny of Hyaloperonospora based on nuclear ribosomal internal transcribed spacer sequences. Mycol. Prog. 3, 83–94. 10.1007/s11557-006-0079-7

[B22] Goritschnig S. Krasileva K. V. Dahlbeck D. Staskawicz B. J. (2012). Computational prediction and molecular characterization of an oomycete effector and the cognate Arabidopsis resistance gene. PLoS Genet. 8:e1002502. 10.1371/journal.pgen.1002502 22359513 PMC3280963

[B23] Hall B. G. Yokoyama S. Calhoun D. H. (1983). Role of cryptic genes in microbial evolution. Mol. Biol. Evol. 1, 109–124. 6400646 10.1093/oxfordjournals.molbev.a040300

[B24] Halterman D. Chen Y. Sopee J. Berduo-Sandoval J. Sanchez-Pérez A. (2010). Competition between *Phytopthora infestans* leads to increase agressiveness containing broad spectrum late-blight resistance. PLoS ONE 5:e10536. 10.1371/journal.pone.0010536 20479869 PMC2866322

[B25] Hedrick P. W. (2012). What is the evidence for heterozygote advantage selection? Trends Ecol. Evol. 27, 698–704. 10.1016/j.tree.2012.08.012 22975220

[B26] Hewezi T. (2015). Cellular signaling pathways and posttranslational modifications mediated by nematode effector proteins. Plant Physiol. 169, 1018–1026. 10.1104/pp.15.00923 26315856 PMC4587465

[B27] Holub E. B. (2006). Evolution of parasitic symbiosis between plants and filamentous microorganisms. Curr. Opin. Plant Biol. 9, 397–405. 10.1016/j.pbi.2006.05.011 16714140

[B28] Holub E. B. Beynon J. L. Crute I. R. (1994). Phenotypic and genotypic character-ization of interactions between isolates of *Peronospora parasitica* and accessions of *Arabidopsis thaliana*. Mol. Plant Microbe Interact. 7, 223–239. 10.1094/MPMI-7-0223

[B29] Houterman P. M. Cornelissen B. J. Rep M. (2008). Suppression of plant resistance gene-based immunity by a fungal effector. PLoS Pathog. 4:e1000061. 10.1371/journal.ppat.1000061 18464895 PMC2330162

[B30] Jones J. D. Dangl J. L. (2006). The plant immune system. Nature 444, 323–329. 10.1038/nature05286 17108957

[B31] Kamoun S. (2006). A catalogue of the effector secretome of plant pathogenic oomycetes. Annu. Rev. Phytopathol. 44, 41–60. 10.1146/annurev.phyto.44.070505.143436 16448329

[B32] Kamoun S. Furzer O. Jones J. D. Judelson H. S. Ali G. S. Dalio R. J.. (2015). The Top 10 oomycete pathogens in molecular plant pathology. Mol. Plant Pathol. 16, 413–434 10.1111/mpp.12190 25178392 PMC6638381

[B33] Kearse M. Moir R. Wilson A. Stones-Havas S. Cheung M. Sturrock S.. (2012). Geneious Basic, an integrated and extendable desktop software platform for the organization and analysis of sequence data. Bioinformatics 28, 1647–1649. 10.1093/bioinformatics/bts199 22543367 PMC3371832

[B34] Keeling P. J. Burger G. Durnford D. G. Lang B. F. Lee R. W. Pearlman R. E.. (2005). The tree of eukaryotes. Trends Ecol. Evol. 20, 670–676. 10.1016/j.tree.2005.09.005 16701456

[B35] Kelley L. A. Mezulis S. Yates C. M. Wass M. N. Sternberg M. J. (2015). The Phyre2 web portal for protein modeling, prediction and analysis. Nat. Protoc. 10, 845–858. 10.1038/nprot.2015.053 25950237 PMC5298202

[B36] Koch E. Slusarenko A. (1990). Arabidopsis is susceptible to infection by a downy mildew fungus. Plant Cell 2, 437–445. 10.1105/tpc.2.5.437 2152169 PMC159900

[B37] Kolmer J. A. (1992). Virulence heterozygosity and gametic phase disequilibria in two populations of *Puccinia recondita* (wheat leaf rust fungus). Heredity 68, 505–513. 10.1038/hdy.1992.73

[B38] Kong Y. (2011). Btrim, a fast, lightweight adapter and quality trimming program for next-generation sequencing technologies. Genomics 98, 152–153. 10.1016/j.ygeno.2011.05.009 21651976

[B39] Krasileva K. V. Zheng C. Leonelli L. Goritschnig S. Dahlbeck D. Staskawicz B. J. (2011). Global analysis of Arabidopsis/Downy mildew interactions reveals prevalence of incomplete resistance and rapid evolution of pathogen recognition. PLoS ONE 6:e28765. 10.1371/journal.pone.0028765 22194907 PMC3237489

[B40] Krogh A. Larsson B. von Heijne G. Sonnhammer E. L. (2001). Predicting transmembrane protein topology with a hidden Markov model, application to complete genomes. J. Mol. Biol. 305, 567–580. 10.1006/jmbi.2000.4315 11152613

[B41] Lamour K. H. Mudge J. Gobena D. Hurtado-Gonzales O. P. Schmutz J. Kuo A.. (2012). Genome sequencing and mapping reveal loss of heterozygosity as a mechanism for rapid adaptation in the vegetable pathogen *Phytophthora capsici*. Mol. Plant Microbe Interact. 25, 1350–1360. 10.1094/MPMI-02-12-0028-R 22712506 PMC3551261

[B42] Lau G. W. Chao C. T. Ellingboe A. H. (1993). Interaction of genes controlling avirulence/virulence of *Magnaporthe grisea* on rice cultivar Katy. Phytopathology 83, 375–382. 10.1094/Phyto-83-375

[B43] Lawrence G. J. Mayo G. M. E. Shepherd K. W. (1981). Interactions between genes controlling pathogenicity in the flax rust fungus. Phytopathology 71, 12–19. 10.1094/Phyto-71-12

[B44] Le Gac M. Hood M. E. Fournier E. Giraud T. (2007). Phylogenetic evidence of host-specific cryptic species in the anther smut fungus. Evolution 61, 15–26. 10.1111/j.1558-5646.2007.00002.x 17300424

[B45] Lee S. Garzó C. D. Moorman G. W. (2010). Genetic structure and distribution of *Pythium aphanidermatum* populations in Pennsylvania greenhouses based on analysis of AFLP and SSR markers. Mycologia 102, 774–784. 10.3852/09-018 20648746

[B46] Leonelli L. Pelton J. Schoeffler A. Dahlbeck D. Berger J. Wemmer D. E.. (2011). Structural elucidation and functional characterization of the *Hyaloperonospora arabidopsidis* effector protein ATR13. PLoS Pathog. 7:e1002428. 10.1371/journal.ppat.1002428 22194684 PMC3240608

[B47] Letunic I. Doerks T. Bork P. (2012). SMART 7, recent updates to the protein domain annotation resource. Nucleic Acids Res. 40, D302–D305. 10.1093/nar/gkr931 22053084 PMC3245027

[B48] Li W. H. (1984). Retention of cryptic genes in microbial populations. Mol. Biol. Evol. 1, 213–219. 6599964 10.1093/oxfordjournals.molbev.a040312

[B49] Li Y. van der Lee T. A. Evenhuis A. van den Bosch G. B. van Bekkum P. J. Förch M. G.. (2012). Population dynamics of *Phytophthora infestans* in the Netherlands reveals expansion and spread of dominant clonal lineages and virulence in sexual offspring. G3 2, 1529–1540. 10.1534/g3.112.004150 23275876 PMC3516475

[B50] Livak K. J. Schmittgen T. D. (2001). Analysis of relative gene expression data using real-time quantitative PCR and the 2(−^ΔΔ^C(T)) method. Methods 25, 402–408. 10.1006/meth.2001.1262 11846609

[B51] Luzzatto L. (2012). Sickle cell anaemia and malaria. Mediterr. J. Hematol. Infect. Dis. 4:e2012065. 10.4084/mjhid.2012.065 23170194 PMC3499995

[B52] McDowell J. M. Dhandaydham M. Long T. A. Aarts M. G. Goff S. Holub E. B.. (1998). Intragenic recombination and diversifying selection contribute to the evolution of downy mildew resistance at the RPP8 locus of Arabidopsis. Plant Cell 10, 1861–1874. 10.1105/tpc.10.11.1861 9811794 PMC143965

[B53] Modiano G. Ciminelli B. M. Pignatti P. F. (2007). Cystic fibrosis and lactase persistence, a possible correlation. Eur. J. Hum. Genet. 15, 255–259. 10.1038/sj.ejhg.5201749 17180122

[B54] Na R. Gijzen M. (2016). Escaping host immunity, New tricks for plant pathogens. PLoS Pathog. 12:e1005631. 10.1371/journal.ppat.1005631 27389195 PMC4936731

[B55] Neff M. M. Turk E. Kalishman M. (2002). Web-based primer design for single nucleotide polymorphism analysis. Trends Genet. 18, 613–615. 10.1016/S0168-9525(02)02820-2 12446140

[B56] Nemri A. Atwell S. Tarone A. M. Huang Y. S. Zhao K. Studholme D. J.. (2010). Genome-wide survey of Arabidopsis natural variation in downy mildew resistance using combined association and linkage mapping. Proc. Natl. Acad. Sci. U.S.A. 107, 10302–10307. 10.1073/pnas.0913160107 20479233 PMC2890483

[B57] Nordborg M. Hu T. T. Ishino Y. Jhaveri J. Toomajian C. Zheng H.. (2005). The pattern of polymorphism in *Arabidopsis thaliana*. PLoS Biol. 3:e196. 10.1371/journal.pbio.0030196 15907155 PMC1135296

[B58] Parker J. E. Holub E. B. Frost L. N. Falk A. Gunn N. D. Daniels M. J. (1996). Characterization of eds1, a mutation in Arabidopsis suppressing resistance to *Peronospora parasitica* specified by several different RPP genes. Plant Cell 8, 2033–2046. 10.1105/tpc.8.11.2033 8953768 PMC161332

[B59] Pedersen C. Ver Loren van Themaat E. McGuffin L. J. Abbott J. C. Burgis T. A. Barton G.. (2012). Structure and evolution of barley powdery mildew effector candidates. BMC Genomics 13:694. 10.1186/1471-2164-13-694 23231440 PMC3582587

[B60] Penn D. J. Damjanovich K. Potts W. K. (2002). MHC heterozygosity confers a selective advantage against multiple-strain infections. Proc. Natl. Acad. Sci. U.S.A. 99, 11260–11264. 10.1073/pnas.162006499 12177415 PMC123244

[B61] Petersen T. N. Brunak S. von Heijne G. Nielsen H. (2011). SignalP 4.0, discriminating signal peptides from transmembrane regions. Nat. Methods 8, 785–786. 10.1038/nmeth.1701 21959131

[B62] Punta M. Coggill P. C. Eberhardt R. Y. Mistry J. Tate J. Boursnell C.. (2012). The Pfam protein families database. Nucleic Acids Res. 40, D290–D301. 10.1093/nar/gkr1065 22127870 PMC3245129

[B63] Quevillon E. Silventoinen V. Pillai S. Harte N. Mulder N. Apweiler R.. (2005). InterProScan, protein domains identifier. Nucleic Acids Res. 33, W116–W120. 10.1093/nar/gki442 15980438 PMC1160203

[B64] Rehmany A. P. Gordon A. Rose L. E. Allen R. L. Armstrong M. R. Whisson S. C.. (2005). Differential recognition of highly divergent downy mildew avirulence gene alleles by RPP1 resistance genes from two Arabidopsis lines. Plant Cell 17, 1839–1850. 10.1105/tpc.105.031807 15894715 PMC1143081

[B65] Shan L. He P. Li J. Heese A. Peck S. C. Nürnberger T.. (2008). Bacterial effectors target the common signaling partner BAK1 to disrupt multiple MAMP receptor-signaling complexes and impede plant immunity. Cell Host Microbe 4, 17–27. 10.1016/j.chom.2008.05.017 18621007 PMC2830012

[B66] Sinapidou E. Williams K. Nott L. Bahkt S. Tör M. Crute I.. (2004). Two TIR: NB:LRR genes are required to specify resistance to Peronospora parasitica isolate Cala_2_ in Arabidopsis. Plant J. 38, 898–909. 10.1111/j.1365-313X.2004.02099.x 15165183

[B67] Sohn K. H. Lei R. Nemri A. Jones J. D. (2007). The downy mildew effector proteins ATR1 and ATR13 promote disease susceptibility in *Arabidopsis thaliana*. Plant Cell 19, 4077–4090. 10.1105/tpc.107.054262 18165328 PMC2217653

[B68] Song J. Win J. Tian M. Schornack S. Kaschani F. Ilyas M.. (2009). Apoplastic effectors secreted by two unrelated eukaryotic plant pathogens target the tomato defense protease Rcr3. Proc. Natl. Acad. Sci. U.S.A. 106, 1654–1659. 10.1073/pnas.0809201106 19171904 PMC2635833

[B69] Steinbrenner A. D. Goritschnig S. Staskawicz B. J. (2015). Recognition and activation domains contribute to allele-specific responses of an Arabidopsis NLR receptor to an oomycete effector protein. PLoS Pathog. 11:e1004665. 10.1371/journal.ppat.1004665 25671309 PMC4335498

[B70] Tör M. Gordon P. Cuzick A. Eulgem T. Sinapidou E. Mert-Türk F.. (2002). Arabidopsis SGT1b Is required for defense signaling conferred by several downy mildew resistance genes. Plant Cell 14, 993–1003. 10.1105/tpc.001123 12034892 PMC150602

[B71] Tör M. Holub E. B. Brose E. Musker R. Gunn N. (1994). Map positions of three loci in *Arabidopsis thaliana* associated with isolate-specific recognition of *Peronospora parasitica* (downy mildew). Mol. Plant Microbe Interact. 7, 214–222. 10.1094/MPMI-7-0214

[B72] Tyler B. M. Kale S. D. Wang Q. Tao K. Clark H. R. Drews K.. (2013). Microbe-independent entry of oomycete RxLR effectors and fungal RxLR-like effectors into plant and animal cells is specific and reproducible. Mol. Plant Microbe Interact. 26, 611–616. 10.1094/MPMI-02-13-0051-IA 23550528 PMC3994703

[B73] van der Biezen E. A. Freddie C. T. Kahn K. Parker J. E. Jones J. D. (2002). Arabidopsis RPP4 is a member of the RPP5 multigene family of TIR-NB-LRR genes and confers downy mildew resistance through multiple signalling components. Plant J. 29, 439–451. 10.1046/j.0960-7412.2001.01229.x 11846877

[B74] Vinatzer B. A. Teitzel G. M. Lee M. W. Jelenska J. Hotton S. Fairfax K.. (2006). The type III effector repertoire of *Pseudomonas syringae* pv. syringae B728a and its role in survival and disease on host and non-host plants. Mol. Microbiol. 62, 26–44. 10.1111/j.1365-2958.2006.05350.x 16942603

[B75] Wawra S. Trusch F. Matena A. Apostolakis K. Linne U. Zhukov I.. (2017). The RxLR motif of the host targeting effector AVR3a of *Phytophthora infestans* is cleaved before secretion. Plant Cell 29, 1184–1195. 10.1105/tpc.16.00552 28522546 PMC5502441

[B76] Whisson S. C. Boevink P. C. Moleleki L. Avrova A. O. Morales J. G. Gilroy E. M.. (2007). A translocation signal for delivery of oomycete effector proteins into host plant cells. Nature 450, 115–118. 10.1038/nature06203 17914356

[B77] Whitford R. Fleury D. Reif J. C. Garcia M. Okada T. Korzun V.. (2013). Hybrid breeding in wheat, technologies to improve hybrid wheat seed production. J. Exp. Bot. 64, 5411–5428. 10.1093/jxb/ert333 24179097

[B78] Win J. Chaparro-Garcia A. Belhaj K. Saunders D. G. Yoshida K. Dong S.. (2013). Effector biology of plant-associated organisms, concepts and perspectives. Cold Spring Harb. Symp. Quant. Biol. 77, 235–247. 10.1101/sqb.2012.77.015933 23223409

[B79] Zheng W. Huang L. Huang J. Wang X. Chen X. Zhao J.. (2013). High genome heterozygosity and endemic genetic recombination in the wheat stripe rust fungus. Nat. Commun. 4, 2673. 10.1038/ncomms3673 24150273 PMC3826619

