# Supplementary figures and images for: A Suppressor/Avirulence Gene Combination in Hyaloperonospora arabidopsidis Determines Race Specificity in Arabidopsis thaliana
Source: Front Plant Sci. 2018 Mar 1;9:265. doi: 10.3389/fpls.2018.00265 (PMC5838922; doi:10.3389/fpls.2018.00265)

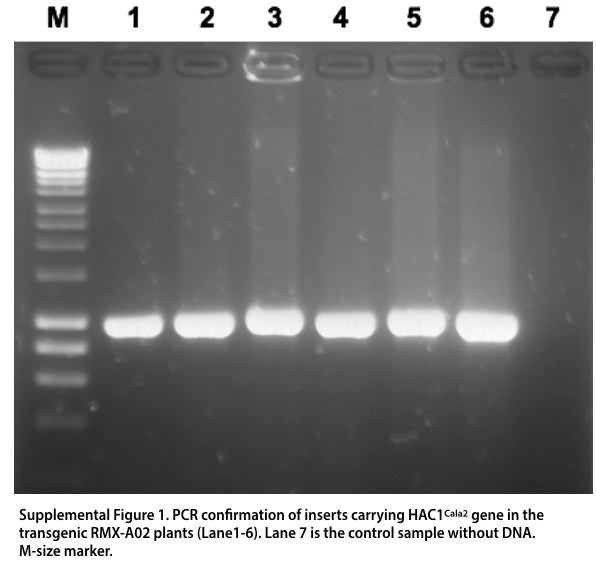

Supplement: Supplemental Figure 1 — PCR confirmation of HAC1Cala2 inserts in transgenic RMX-A02 plants. [file Image1.jpeg]

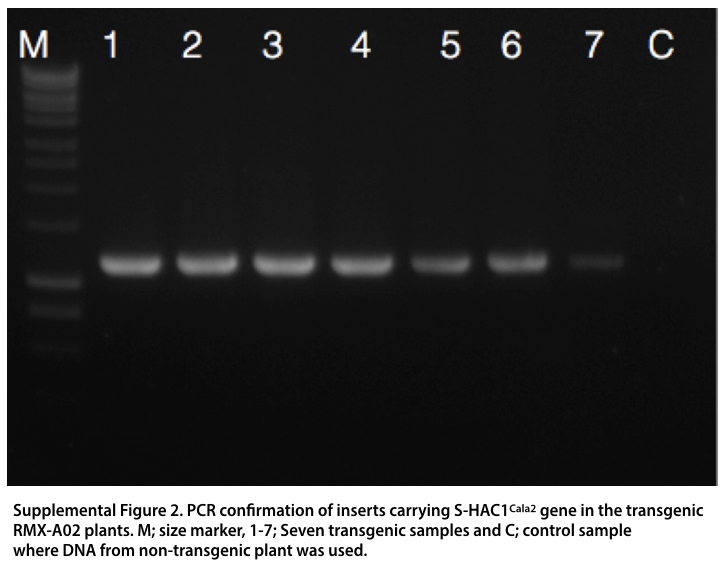

Supplement: Supplemental Figure 2 — PCR confirmation of inserts carrying S-HAC1Cala2 gene in the transgenic RMX-A02 plants. [file Image2.jpeg]

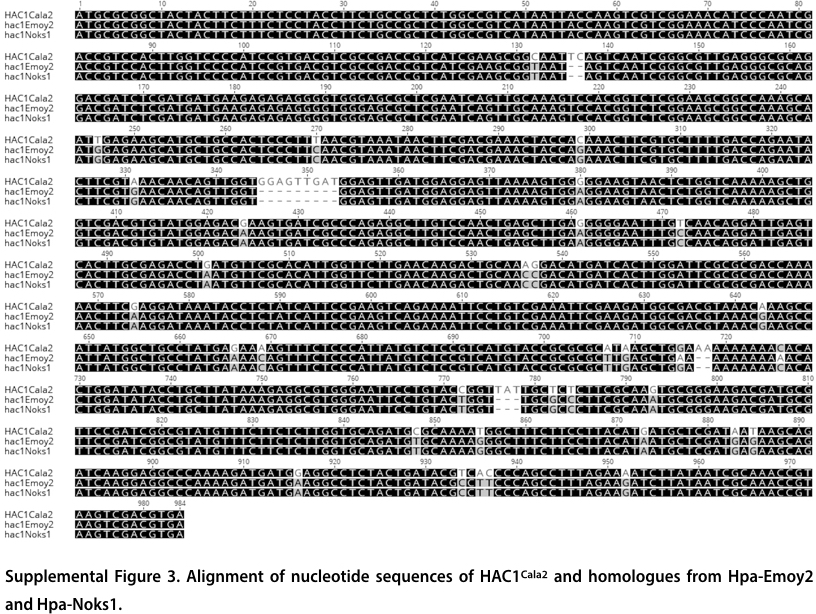

Supplement: Supplemental Figure 3 — Alignment of nucleotide sequences of HAC1Cala2 and homologs from Hpa-Emoy2 and Hpa-Noks1. [file Image3.jpeg]

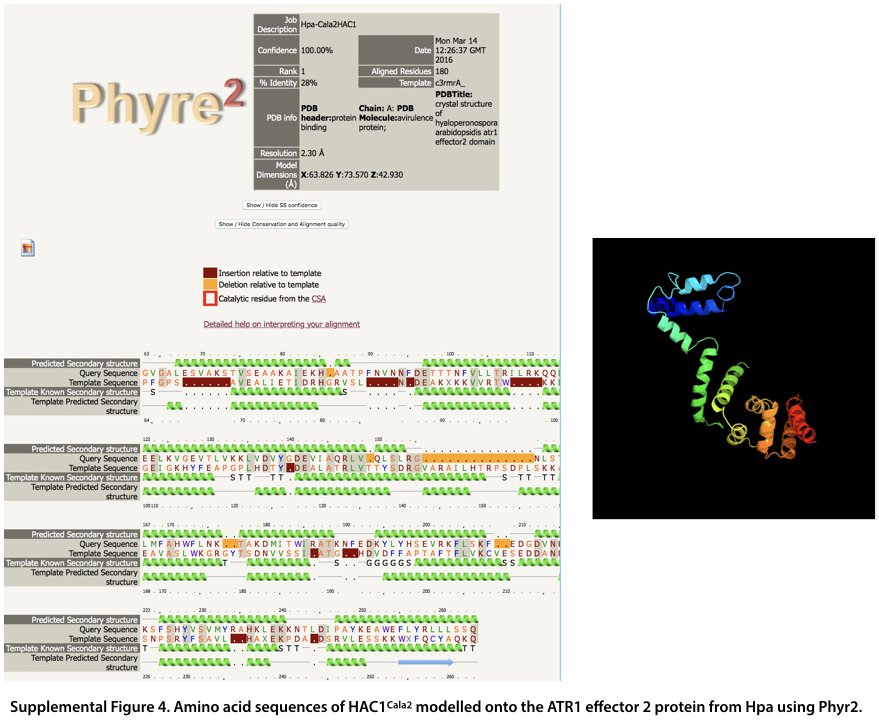

Supplement: Supplemental Figure 4 — Amino acid sequences of HAC1Cala2 modeled onto the ATR1 effector 2 protein from Hpa using Phyr2. [file Image4.jpeg]

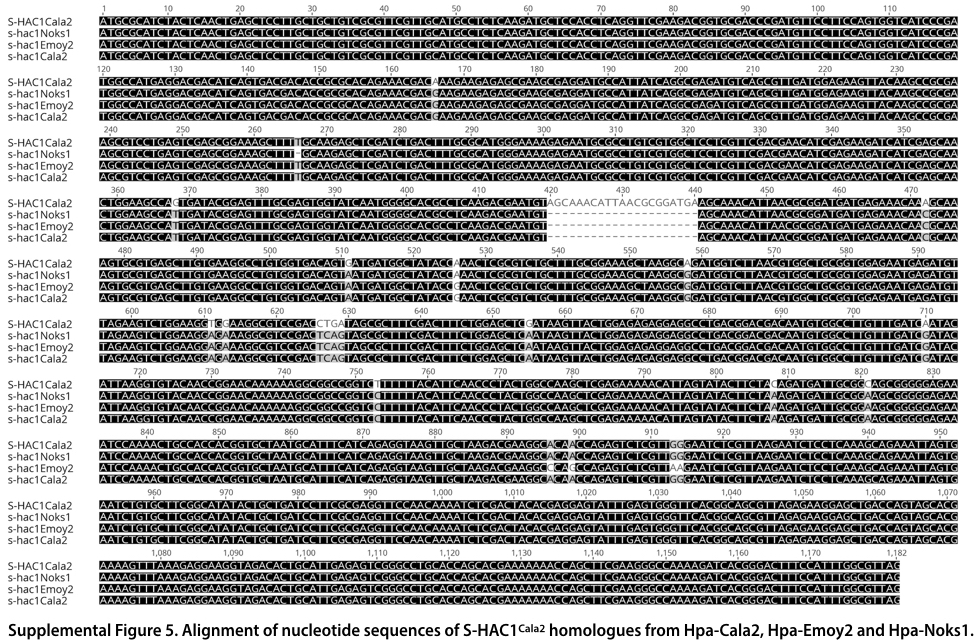

Supplement: Supplemental Figure 5 — Alignment of nucleotide sequences of S-HAC1Cala2 homologs from Hpa-Cala2, Hpa-Emoy2, and Hpa-Noks1. [file Image5.jpeg]

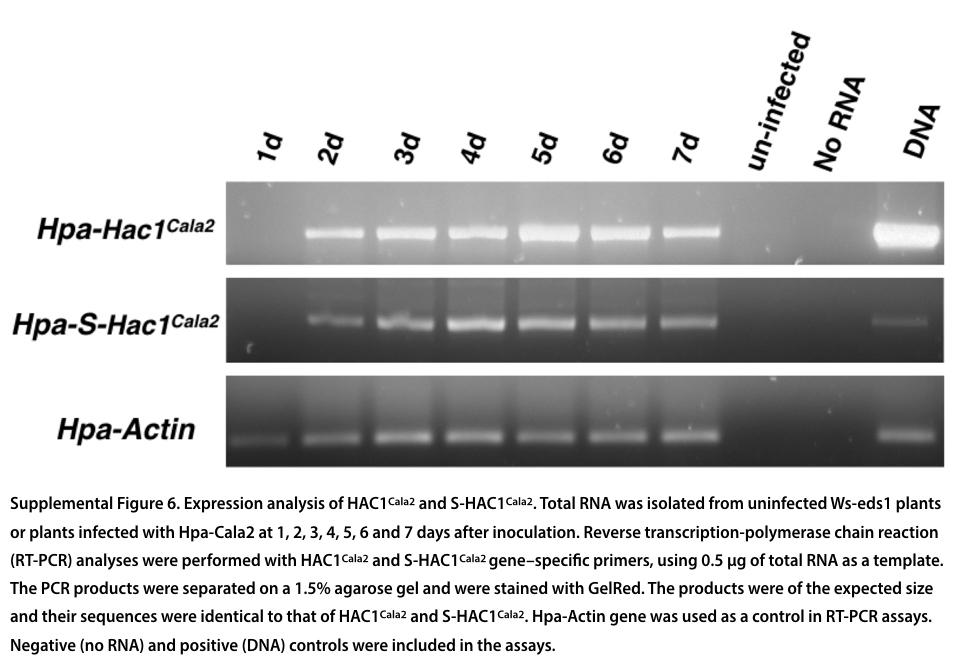

Supplement: Supplemental Figure 6 — Expression analysis of HAC1Cala2 and S-HAC1Cala2. [file Image6.jpeg]
